# Histone lysine demethylase 4B regulates general and unique gene expression signatures in hypoxic cancer cells

**DOI:** 10.1002/mco2.85

**Published:** 2021-08-27

**Authors:** Lei Qiu, Yang Meng, Lingli Wang, Sumedha Gunewardena, Sicheng Liu, Junhong Han, Adam J. Krieg

**Affiliations:** ^1^ Research Laboratory of Cancer Epigenetics and Genomics Department of General Surgery Frontiers Science Center for Disease‐related Molecular Network Cancer Center West China Hospital Sichuan University Chengdu China; ^2^ Department of Obstetrics and Gynecology University of Kansas Medical Center Kansas City Kansas USA; ^3^ Department of Pathology and Laboratory Medicine University of Kansas Medical Center Kansas City Kansas USA; ^4^ Department of Molecular and Integrative Physiology University of Kansas Medical Center Kansas City Kansas USA; ^5^ Department of Obstetrics and Gynecology Oregon Health and Science University USA; ^6^ Division of Reproductive and Developmental Sciences Oregon National Primate Research Center Beaverton Oregon USA

**Keywords:** histone lysine demethylase 4B, colorectal cancer, ovarian cancer, renal cell carcinoma, tumor hypoxia

## Abstract

The hypoxic tumor microenvironment promotes tumor survival by inducing the expression of genes involved in angiogenesis and metastasis. As a direct target of hypoxia‐inducible factor, lysine demethylase 4B (*KDM4B*) is overexpressed in multiple cancers, suggesting that a general KDM4B regulatory mechanism may exist in these cancer types. In this study, we sought to further investigate the general and unique roles of KDM4B in ovarian, colon, and renal cancer cells. We first identified a set of potential KDM4B targets shared by SKOV3ip.1, HCT116, and RCC4 cell lines, as well as numerous genes specifically regulated in each cell line. Through Gene Ontology, KEGG, and Oncobox pathway analyses, we found that KDM4B primarily regulated biosynthetic and cell cycle pathways in normoxia, whereas in hypoxia, it regulated pathways associated with inflammatory response and migration. TCGA data analyses reveal high expression of *KDM4B* in multiple cancer types and differential expression across cancer stages. Kaplan–Meier plots suggest that elevated *KDM4B* expression may contribute to a better or worse prognosis in a manner specific to each cancer type. Overall, our findings suggest that *KDM4B* plays complex roles in regulating multiple cancer processes, providing a useful resource for the future development of cancer therapies that target *KDM4B* expression.

## Graphical abstract

KDM4B regulates 26 common genes shared by HCT116, SKOV3ip.1, and RCC4 cell lines.

KDM4B regulates numerous genes that are specific for each cell line.

KDM4B regulates cell cycle‐related targets in normoxia and inflammatory response and migration‐related genes in hypoxia.

KDM4B is highly expressed in multiple cancers and is differentially expressed across cancer stages.

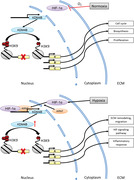

## 1 INTRODUCTION

Hypoxia correlates with poorprognosis for cancer patients and is an important regulator of tumorigenesis by inducing angiogenesis, metastasis, and glycolysis.^1^ The hypoxia‐inducible factor (HIF) family of transcription factors are “master regulators” of hypoxic gene expression and directly regulate the expression of genes that promote cancer progression in the following pathways: proliferation, viability, glycolysis, migration, tissue remodeling, and angiogenesis.^2^, ^3^

As a direct downstream target of HIF‐1, lysine demethylase 4B (KDM4B) catalyzes H3K9me3/me2 demethylation, a mechanism associated with gene activation.^4^, ^5^, ^6^, ^7^, ^8^, ^9^ *KDM4B* overexpression has been reported in many cancers, including colon, breast, prostate, and ovarian cancers.^10^, ^11^, ^12^, ^13^ We have previously shown that KDM4B regulates peritoneal seeding of ovarian cancer (OVCAR) by demethylating the promoters of *PDGFB*, *LOX*, *LOXL2*, and *LCN2* genes.^13^ HIF‐1α‐induction of *KDM4B* supports colorectal cancer (CRC) malignancy by regulating metastasis and proliferation‐related genes.^11^ The most commonly known characteristic of clear cell renal cell carcinoma (RCC) is mutation or loss of *von Hippel‐Lindau (VHL)*, leading to constitutively HIF activation.^14^ *KDM4B* overexpression has been reported in RCC,^4^, ^6^ yet its potential contribution to RCC progression remains uncharacterized. Although all of these cancers are located in the peritoneal cavity, they each have distinct clinical phenotypes and molecular regulatory mechanisms. In this study, we sought to determine the role KDM4B plays in these three cancer types, whether it regulates general or unique gene expression signatures and if there is a correlation between KDM4B regulatory mechanism and the hypoxia‐induced HIF signaling.

We first identified KDM4B‐dependent genes by comparing gene expression levels in the OVCAR cell line SKOV3ip.1, CRC cell line HCT116, and the RCC cell line RCC4 after knocking down KDM4B. By using microarray and Venn diagram overlap analyses, we found a list of 26 common KDM4B‐dependent genes, as well as many cell line‐specific genes that are regulated by KDM4B. Among the 26 common targets, 16 genes were positively regulated by KDM4B, possibly through the H3K9me3/me2 demethylase activity of KDM4B. Through comparing KDM4B‐dependent genes in normoxic and hypoxic conditions in SKOV3ip.1 and HCT116 cell lines, we also discovered that KDM4B regulated biosynthesis, cell cycle, and proliferation‐related pathways in normoxic condition, whereas it regulated genes related to stress response, inflammatory response, and migration in hypoxia. Our data demonstrated that KDM4B preferentially regulated distinct pathways in different oxygen microenvironments, suggesting that KDM4B may be an important factor for tumor hypoxia maintenance or promotion.

## 2 RESULTS

### 2.1 KDM4B regulates common and unique gene expression signatures in SKOV3ip.1, HCT116, and RCC4 cell lines

**FIGURE 1:**
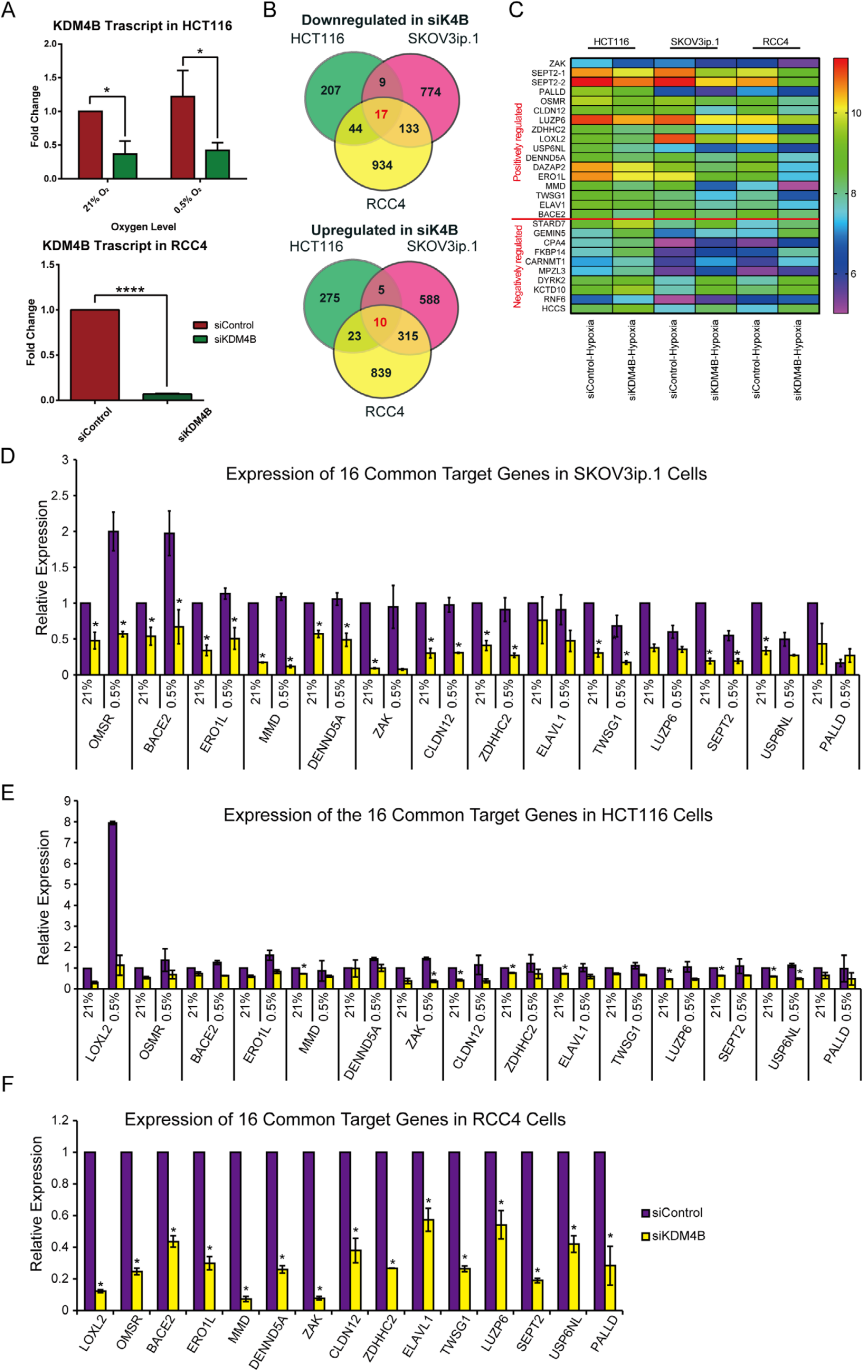
Microarray analysis identified common lysine demethylase 4B (KDM4B)‐dependent genes in SKOV3ip.1, HCT116 and RCC4 cell lines. (A) Quantitative real‐time PCR (QPCR) confirmed *KDM4B* knockdown efficiency in both HCT116 and RCC4 cell lines. (B) Venn diagram demonstrating the number of KDM4B‐dependent target genes (both downregulated by siK4B and upregulated by siK4B) in each cell line as well as common targets shared by the three cell lines. Hypergeometric distribution was used to calculate the statistical significance of each overlap region. Among the genes downregulated by siK4B, *p *< 4.446 × 10^−16^ for overlap between HCT116 and RCC4, *p *< 0.01 for overlap between HCT116 and SKOV3ip.1, *p *< 4.253 × 10^−23^ for overlap between RCC4 and SKOV3ip.1. Among the genes upregulated by siK4B, *p *< 0.294 for overlap between HCT116 and SKOV3ip.1, *p *< 3.126 × 10^−146^ for overlap between RCC4 and SKOV3ip.1, *p *< 0.023 for overlap between HCT116 and RCC4. (C) Heatmap representing unsupervised hierarchical clustering of downregulated gene cDNA expression level in SKOV3ip.1, HCT116, and RCC4 cells transfected with siControl or si*KDM4B* in hypoxia (pseudo‐hypoxia for RCC4 cells). Each column represents the indicated sample (mean for three replicates), each row indicates the cDNA level of one gene. Genes above the red line represent genes that are positively regulated by KDM4B (downregulated in three cell lines with siK4B), whereas genes below the red line are those negatively regulated by KDM4B (upregulated in three cell lines with siK4B). (D) SKOV3ip.1 RNA samples. (E) HCT116 RNA samples. (F) RCC4 RNA samples were reverse transcribed and analyzed with QPCR, with primers specifically targeting the 16 common KDM4B targets downregulated by siK4B. In all QPCR studies above, siK4B, siRNA specifically targeting *KDM4B*; siCon, scrambled siRNA. Data represents mean Fold Change (FC) ± Standard Error of the Means (SEM) normalized to 18S rRNA, calculated relative to siCon in 21% O_2_, *N*≥2, *n* = 3. *, *p* < 0.05, determined by two‐tailed paired Student's t‐test

KDM4B is a key epigenetic factor that plays important roles in multiple cancer types.^15^ To determine whether KDM4B regulates mechanisms that are commonly shared by multiple cancers or that are unique for each cancer type, we identified KDM4B‐dependent genes in SKOV3ip.1, HCT116, and RCC4 cell lines. HCT116 is an epithelial colon carcinoma cell line expressing wild type *TP53*.^16^ SKOV3ip.1 is a *TP53*‐deficient ascites fluid‐derived epithelial ovarian cancer cell line generated from SKOV3 parental cells.^17^, ^18^ RCC4 is a RCC cell line deficient of *VHL*, displaying a “pseudo‐hypoxia” phenotype.^19^ Cells were transfected with siRNAs (siK4B or siCon) with robust knockdown effect in both normoxia (21% O_2_) and hypoxia (0.5% O_2;_ Figure 1A, SKOV3ip.1 knockdown validation previously published in fig. 3B by Wilson et al.^13^). RCC4 cells were only tested in normoxia since its *VHL*‐deficiency leads to constitutively active HIF‐1α.^14^ Microarray analysis identified a total of 590 KDM4B‐dependent gene probes in hypoxic HCT116 cells, among which 277 genes were downregulated, and 313 genes were upregulated by KDM4B knockdown (fold change of ≥1.4 or ≤−1.4, compared to siCon cells, *p* < 0.05, Figure 1B). In hypoxic SKOV3ip.1 cells, 933 of the 1851 KDM4B‐dependent gene probes were downregulated, whereas 918 gene probes were upregulated by KDM4B knockdown (Figure 1B). In the pseudo‐hypoxic RCC4 cells, 1128 of the 2315 KDM4B‐dependent gene probes were downregulated, and 1187 gene probes were upregulated by *KDM4B* knockdown (Figure 1B). Only 27 of all these KDM4B‐dependent probes, two of which represent the same gene *SEPT2*, were commonly shared by all three cell lines (Figure 1C, Table S1).

A majority of these KDM4B‐dependent downstream targets are involved in cancer‐related pathways (Table S1). For example, among KDM4B positively regulated genes, *CLDN12, LUZP6, SEPT2, MMD, OSMR, USP6NL*, and *TWSG1* are involved in tumorigenesis and proliferation, whereas genes related to metastasis include *CLDN212, SEPT2, MMD, LOXL2, OSMR, PALLD, ERO1L*, and *ZDHHC2* (Table S1). Among the genes negatively regulated by KDM4B, *STARD7, CPA4, FKBP14*, and *RNF6* have been shown to regulate cell proliferation and/or metastasis, whereas DYRK2 is a known tumor suppressor (Table S1). A few genes are also involved in chemo‐/radiotherapy resistance, including *CLDN12*, *LOXL2*, *OSMR*, and *ZAK* (Table S1). Since the primary function of KDM4B is to activate transcription by demethylating H3K9me3/me2^5^, we validated the KDM4B positively regulated genes with quantitative real‐time PCR (QPCR). Our results demonstrated that *KDM4B* knockdown significantly disrupted the expression of these targets, supporting the results from the microarray analysis (Figure 1D–F, QPCR validation of *LOXL2* gene expression in SKOV3ip.1 was previously published by Wilson et al.^13^).

Besides the 26 genes commonly regulated by KDM4B in three cell lines, more common KDM4B targets were shared by SKOV3ip.1 and RCC4 cells, 113 positively regulated genes and 315 negatively regulated genes (Figure 1B, Gene Ontology (GO) pathway analysis shown in Figure S1). HCT116 shared fewer common targets with each of the other two cell lines, where 44 positively regulated genes and 23 negatively regulated genes were shared by HCT116 and RCC4 cells and only nine positively regulated genes and five negatively regulated genes were common between HCT116 and SKOV3ip.1 cells (Figure 1B), suggesting that KDM4B functional mechanism may be more similar in OVCAR and RCC rather than colon cancer. The majority of KDM4B targets were specific for each cell line (774 positive/588 negative for SKOV3ip.1, 207 positive/275 negative for HCT116, and 934 positive/839 negative for RCC4), suggesting that KDM4B also regulate unique gene expression signatures in each cancer type.

### 2.2 KDM4B regulates distinct pathways in SKOV3ip.1 cells under different oxygen conditions

**FIGURE 2:**
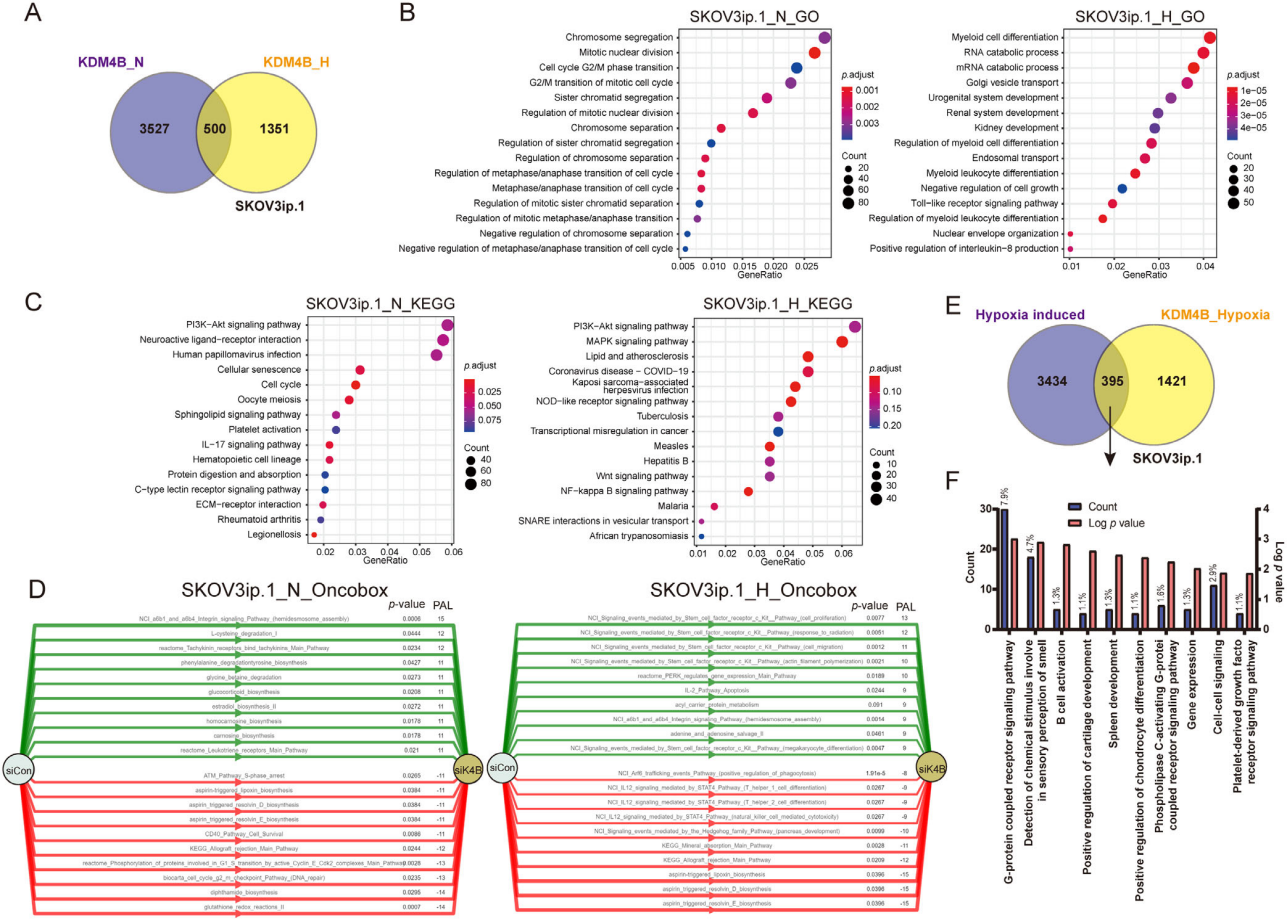
Pathway enrichment analyses for SKOV3ip.1 cells. (A) Venn Diagram showing the number of KDM4B target genes (up‐ and downregulated by siK4B) overlapped in SKOV3ip.1 cells in normoxia and hypoxia. The statistical significance of the overlap region was calculated using hypergeometric distribution, *p *< 8.404 × 10^−41^. (B) KDM4B targets in SKOV3ip.1 cells in normoxia and hypoxia were analyzed using enrichGO from the “clusterProfiler” R package. (C) KDM4B targets in SKOV3ip.1 cells in normoxia and hypoxia were analyzed using enrichKEGG from the “clusterProfiler” R package. (D) KDM4B targets in SKOV3ip.1 cells in normoxia and hypoxia were analyzed using the Oncobox online analytic tool. The top 10 up‐ and downregulated pathways (si*KDM4B* compared to siControl) were shown as green and red stripes with arrows, respectively. PAL, pathway activation level. (E) Venn diagram demonstrating the overlap between genes induced in hypoxia and genes up‐ or downregulated by siK4B in hypoxia in SKOV3ip.1 ovarian cancer cells, *p *< 5.589 × 10^−10^. The statistical significance of each overlap region was calculated using hypergeometric distribution. (F) Gene Ontology (GO) analysis of biological pathways with the overlapped genes from panel in (E)

Although a total of 500 KDM4B‐dependent genes were shared between both hypoxic and normoxic SKOV3ip.1 cells (Figure 2A, both KDM4B positively and negatively regulated genes included), pathway analyses suggested that KDM4B regulated distinct pathways in different oxygen conditions, which supports our previous report.^13^ GO pathway analysis indicated that KDM4B primarily regulated proliferation and cell cycle‐related pathways in normoxia, whereas it regulated pathways functioning in differentiation and tissue development in hypoxia (Figure 2B). When analyzed with the Kyoto Encyclopedia of Genes and Genomes (KEGG) pathway analysis tool, the PI3K‐Akt signaling pathway was regulated by KDM4B in both normoxia and hypoxia (Figure 2C). In normoxia, cellular senescence and cell cycle were listed among the most significantly regulated pathways, supporting that KDM4B played important roles in cell proliferation in normoxia (Figure 2C, left panel). In hypoxia, mitogen‐activated protein kinase (MAPK) signaling was one of the most significantly affected KDM4B‐dependent pathways. The MAPK signaling pathway is essential in regulating many cellular processes including inflammation, stress response, differentiation, proliferation, and apoptosis.^20^ Wnt and NF‐κB signaling pathways are also important inflammatory response pathways that were significantly dysregulated by KDM4B knockdown in hypoxia (Figure 2C, right panel). Interestingly, KDM4B regulated genes implicated in the coronavirus disease (COVID‐19) pathway, including *MAP3K7, MAPK9, IRAK4, IL1B, MYD88, CXCL8, NFKBIA, IKBKE, IFNA7, IFNA5, C3, CFD, C5AR1*, and *TLR3*,^21^, ^22^ as well as multiple ribosome proteins (Figure 2C, right panel, data not shown). These genes are associated with regulating the immune response when threatened with pathogens,^23^ suggesting hypoxic induction of KDM4B may be an important regulator in COVID‐19 immune response in hypoxia.

We thus further calculated the pathway activation levels (PALs) with the Oncobox library, which is a bioinformatic tool that distinguishes functional roles of the pathway ingredients and apply them to annotate 3044 human molecular pathways for the comprehensive analysis of the pathway activation profiles^24^ (Figure 2D). Analysis of PALs clearly showed that genes both positively (red) and negatively (green) regulated by KDM4B in normoxia belonged to pathways related to biosynthesis and cell cycle (Figure 2D, left panel). In contrast, genes regulated by KDM4B in hypoxia were closely associated with stress response, migration, and inflammatory response (Figure 2D, right panel).

Since *KDM4B* is a direct target of HIF,^4^, ^6^, ^7^, ^8^ we sought to know whether KDM4B in turn regulated genes involved in the HIF signaling pathway. Among the 16 genes positively regulated by KDM4B in all three cell lines, 13 genes have been shown to be regulated by hypoxia (Table S2), indicating that KDM4B may be an important regulator in hypoxia‐induced stress response and HIF signaling pathway. We thus compared hypoxia‐induced genes with KDM4B targets in the hypoxic condition in SKOV3ip.1 cell lines and found that a large proportion of genes regulated by KDM4B in hypoxia (78.2%) were not hypoxia‐inducible, suggesting that KDM4B may regulate genes as a secondary effect of HIF signaling, or other compensatory or inhibitory regulators might be involved. There were 395 KDM4 B‐dependent genes (27.8%) induced by hypoxia (Figure 2E). These genes were primarily involved in the inflammatory response, cell movement, and cellular development‐related pathways (Figure 2F). These results suggest that KDM4B may be important for many aspects of cellular function, especially as a regulator of important cancer‐related pathways in different oxygen conditions.

### 2.3 KDM4B regulates multiple cancer‐related pathways in SKOV3ip.1 cells

**FIGURE 3:**
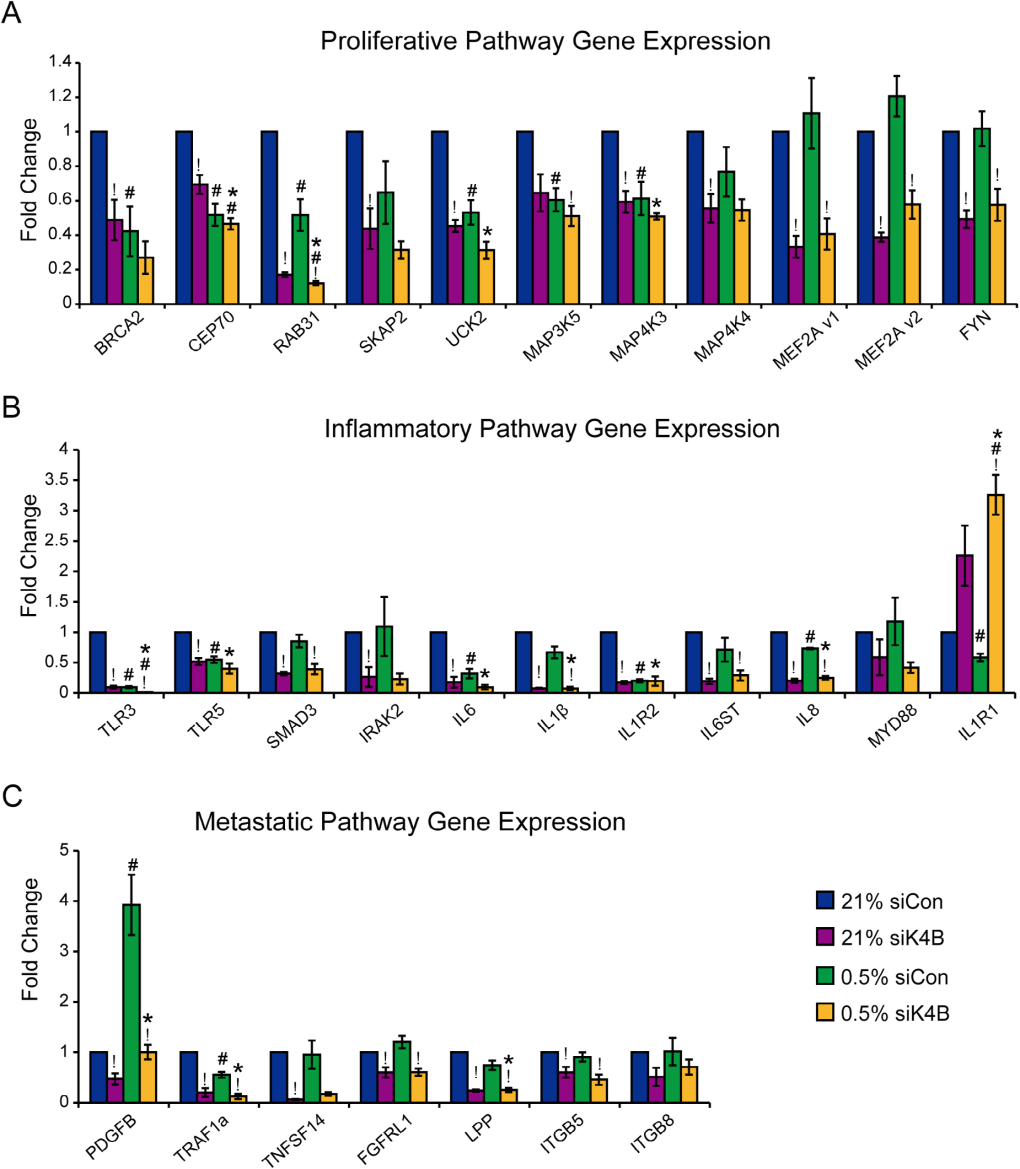
KDM4B regulates genes involved in proliferative, inflammatory, and metastatic pathways in SKOV3ip.1 cells. QPCR validation of selected (A) proliferation‐related genes, (B) inflammation‐related genes, and (C) metastasis‐related genes that are downregulated by siK4B. Data represent mean FC ± SEM, *N* = 3, *n* = 3. Significance of differences was determined using the two‐tailed paired Student's *t*‐test (#, *p* < 0.05 for difference between hypoxia and normoxia; !, *p *< 0.05 for difference between siK4B vs siCon) or using two‐way (*, *p* < 0.05 for two‐way comparison of difference)

In order to better understand the contribution of KDM4B to ovarian cancer progression, pathways regulated by KDM4B in normoxia and hypoxia were analyzed by QPCR (Figure 3). Proliferative genes (*SKAP2, BRCA2, MAP4K3, CEP70 and MAP4K4*) showed robust KDM4B dependency in normoxic conditions, with lesser effects in hypoxia (0.5% siCon; Figure 3A). Many inflammation‐associated genes (*SMAD3, IL‐8, IL‐1B*, and *IL‐6ST*) displayed robust KDM4B dependency in both oxygen conditions (Figure 3B). Additionally, several other genes associated with metastatic pathways (*TRAF1a, PDGFB, LPP, FGFRL1*, and *ITGB5*) were also KDM4B‐dependent in both normoxia and hypoxia (Figure 3C). These data suggest that KDM4B regulates multiple important pathways together to affect ovarian cancer progression.

### 2.4 KDM4B regulates common and distinct pathways in HCT116 cells under different oxygen conditions

**FIGURE 4:**
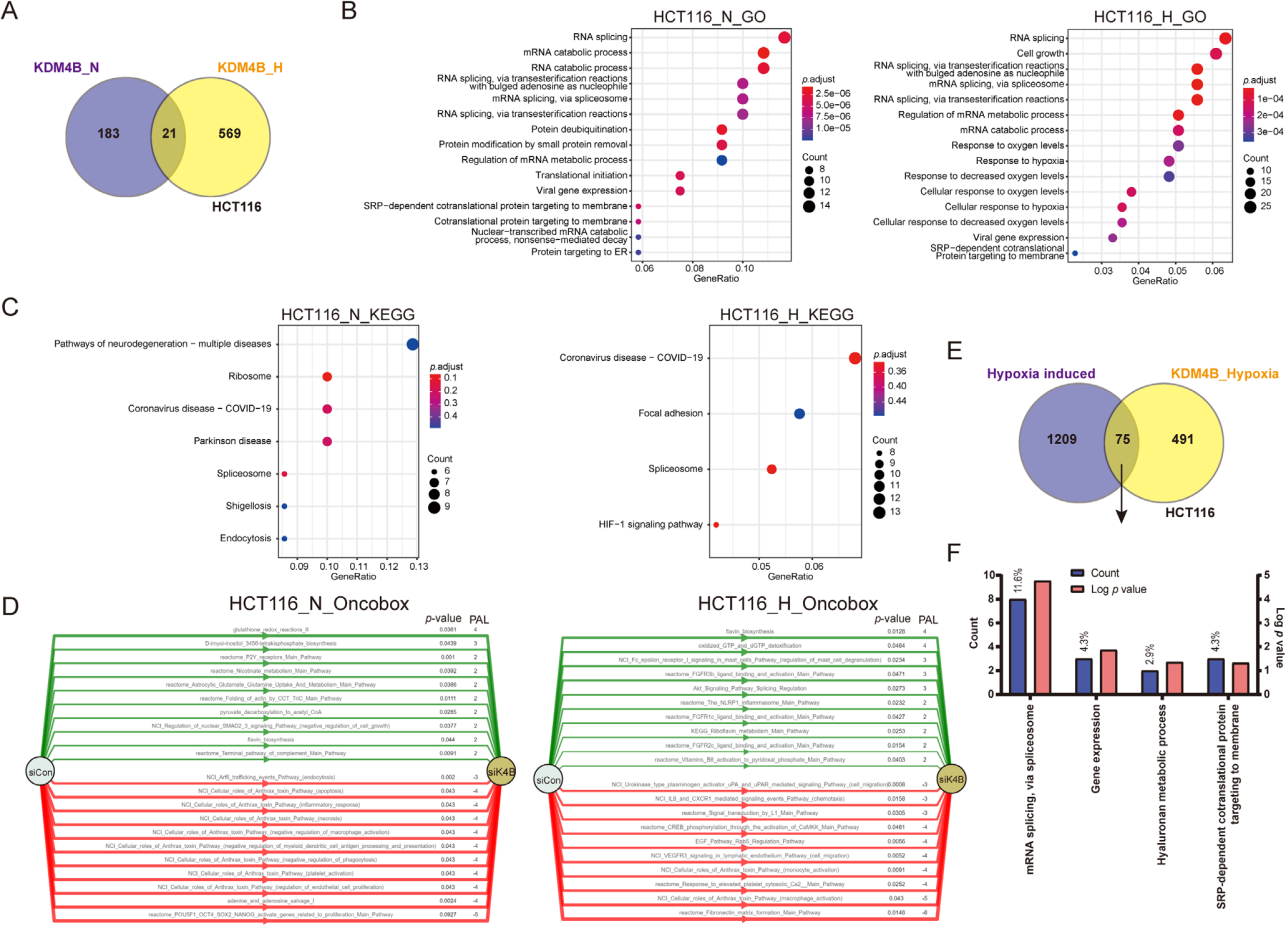
Pathway analyses of KDM4B targets in HCT116 cells. (A) Venn Diagram showing the number of KDM4B target genes (up‐ and downregulated by siK4B) overlapped in HCT116 cells in normoxia and hypoxia. The statistical significance of the overlap region was calculated using hypergeometric distribution, *p *< 1.990 × 10^−6^. (B) KDM4B targets in HCT116 cells in normoxia and hypoxia were analyzed using enrichGO from the “clusterProfiler” R package. (C) KDM4B targets in HCT116 cells in normoxia and hypoxia were analyzed using enrichKEGG from the “clusterProfiler” R package. (D) KDM4B targets in HCT116 cells in normoxia and hypoxia were analyzed using the Oncobox online analytic tool. The top 10 up‐ and downregulated pathways (si*KDM4B*, compared to siControl) were shown as green and red stripes with arrows, respectively. PAL, pathway activation level. (E) Venn diagram demonstrating the overlap between genes induced in hypoxia and genes up‐ or downregulated by siK4B in hypoxia in HCT116 colon cancer cells, *p *< 2.063 × 10^−9^. The statistical significance of each overlap region was calculated using hypergeometric distribution. (F) GO analysis of biological pathways with the overlapped genes from panel in (E)

Although only 21 genes were commonly regulated by KDM4B in normoxia and hypoxia in HCT116 cells (Figure 4A), KDM4B influenced several pathways that are similar in hypoxia and normoxia (Figure 4B and C). When analyzed with the GO pathway analysis tool, the pathway most significantly dysregulated by KDM4B knockdown in normoxia and hypoxia was RNA processing (Figure 4B). KEGG analysis identified only a few signaling pathways that were influenced by KDM4B in each condition (Figure 4C). Interestingly, a COVID‐19‐related pathway appeared in both normoxia (*RPS25, RPS24, RPL9, RPS27A, RPL28, RPS7*, and *NRP1*) and hypoxia (*STAT2, EGFR, NRP1, PRKCA, RPS27A, RPL37A, RPL6, RPL17‐C18orf32, RPS2, RPS5, RPL19, RPL27*, and *RPL11*), further supporting that KDM4B may play crucial roles in the immune response against COVID‐19 infection. When microarray data from HCT116 cells were analyzed with the Oncobox library, KDM4B regulated pathways related to cell growth, biosynthesis, and inflammation in normoxia, whereas it regulated migration‐related pathways in hypoxia (Figure 4D), further supporting that KDM4B may be a crucial factor in cancer progression.

Consistent with observations in SKOV3ip.1 cells, KDM4B regulated only 6.2% of the hypoxia‐inducible genes in HCT116 cells (Figure 4E). Only 75 KDM4B targets (15.3%) were induced by hypoxia in HCT116 cells (Figure 4E), and these genes were primarily involved in fundamental cellular functions, such as mRNA splicing, gene expression, Hyaluronan metabolic process, and protein targeting (Figure 4F). These results further support our previous data in SKOV3ip.1, where KDM4B regulated distinct pathways in different oxygen conditions, suggesting a critical role of KDM4B in cancer progression and fundamental cellular functions.

### 2.5 KDM4B regulates both proliferative and HIF signaling pathways in RCC4 pseudo‐hypoxic cells

**FIGURE 5:**
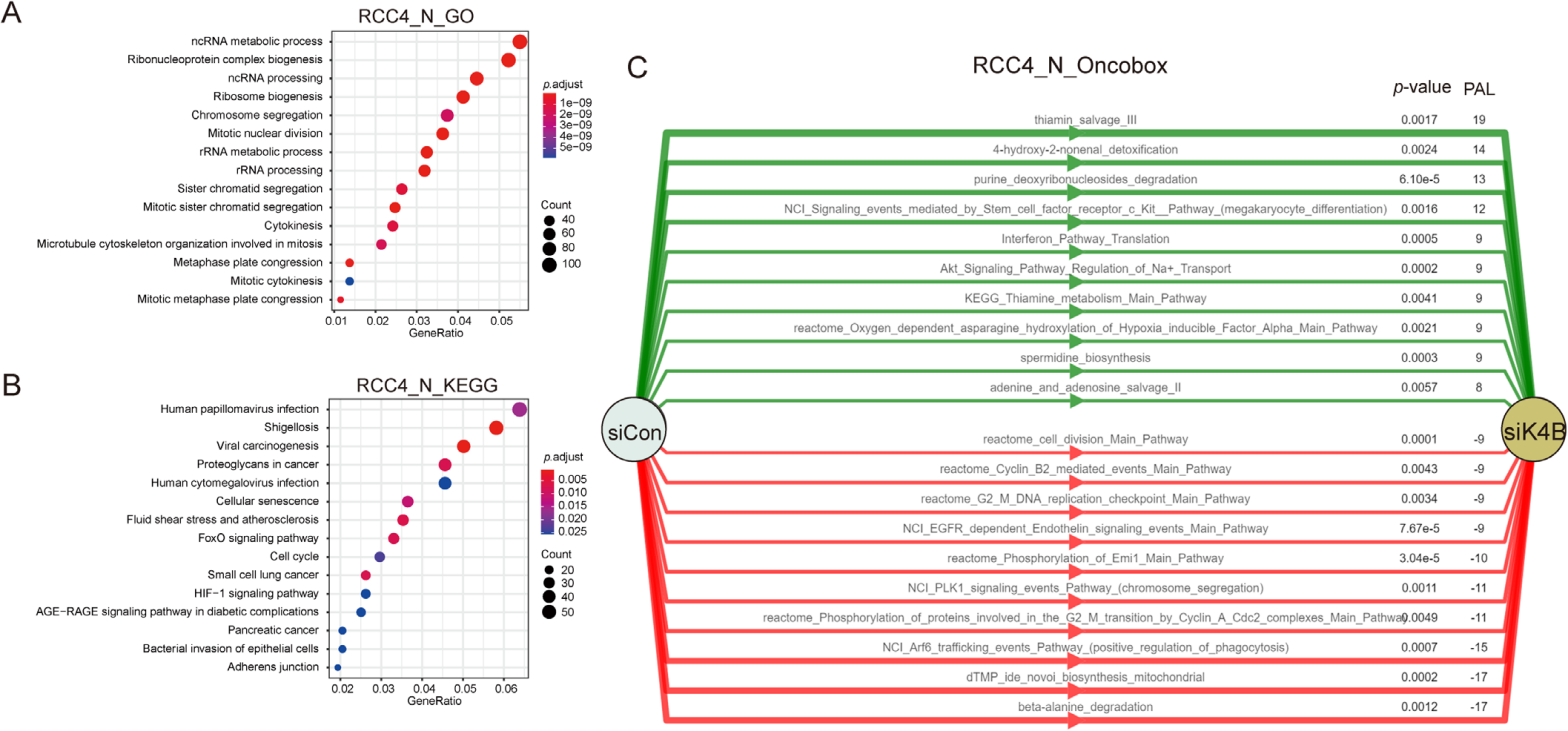
Pathway analyses of KDM4B targets in RCC4 cells. (A) KDM4B targets in RCC4 cells in normoxia were analyzed using enrichGO from the “clusterProfiler” R package. (B) KDM4B targets in RCC4 cells in normoxia were analyzed using enrichKEGG from the “clusterProfiler” R package. (C) KDM4B targets in RCC4 cells in normoxia were analyzed using the Oncobox online analytic tool. The top 10 up‐ and downregulated pathways (si*KDM4B*, compared to siControl) were shown as green and red stripes with arrows, respectively. PAL, pathway activation level

RCC4 cells exhibit a pseudo‐hypoxic phenotype even when cultured in normoxia since the cell line lacks VHL, which ubiquitylates HIF1‐α for degradation in normoxic condition.^14^ When analyzed with the GO analysis tool, KDM4B‐regulated genes belonged primarily to pathways related to cell division and RNA processing (Figure 5A). In KEGG analysis, cellular senescence and cell cycle were significantly regulated pathways (Figure 5B), suggesting KDM4B played important roles in cell proliferation. KEGG analysis also identified the HIF‐1 signaling pathway, suggesting that direct regulation of KDM4B by HIF‐1α provides a mechanism to reinforce HIF‐dependent gene expression in the pseudo‐hypoxic condition created by loss of *VHL*. The Oncobox library analysis provided supporting evidence that KDM4B regulated both cell division‐related pathways and HIF signaling pathway (Figure 5C), suggesting a critical role for KDM4B in RCC progression.

### 2.6 *KDM4B* is highly expressed in multiple cancer types

**FIGURE 6:**
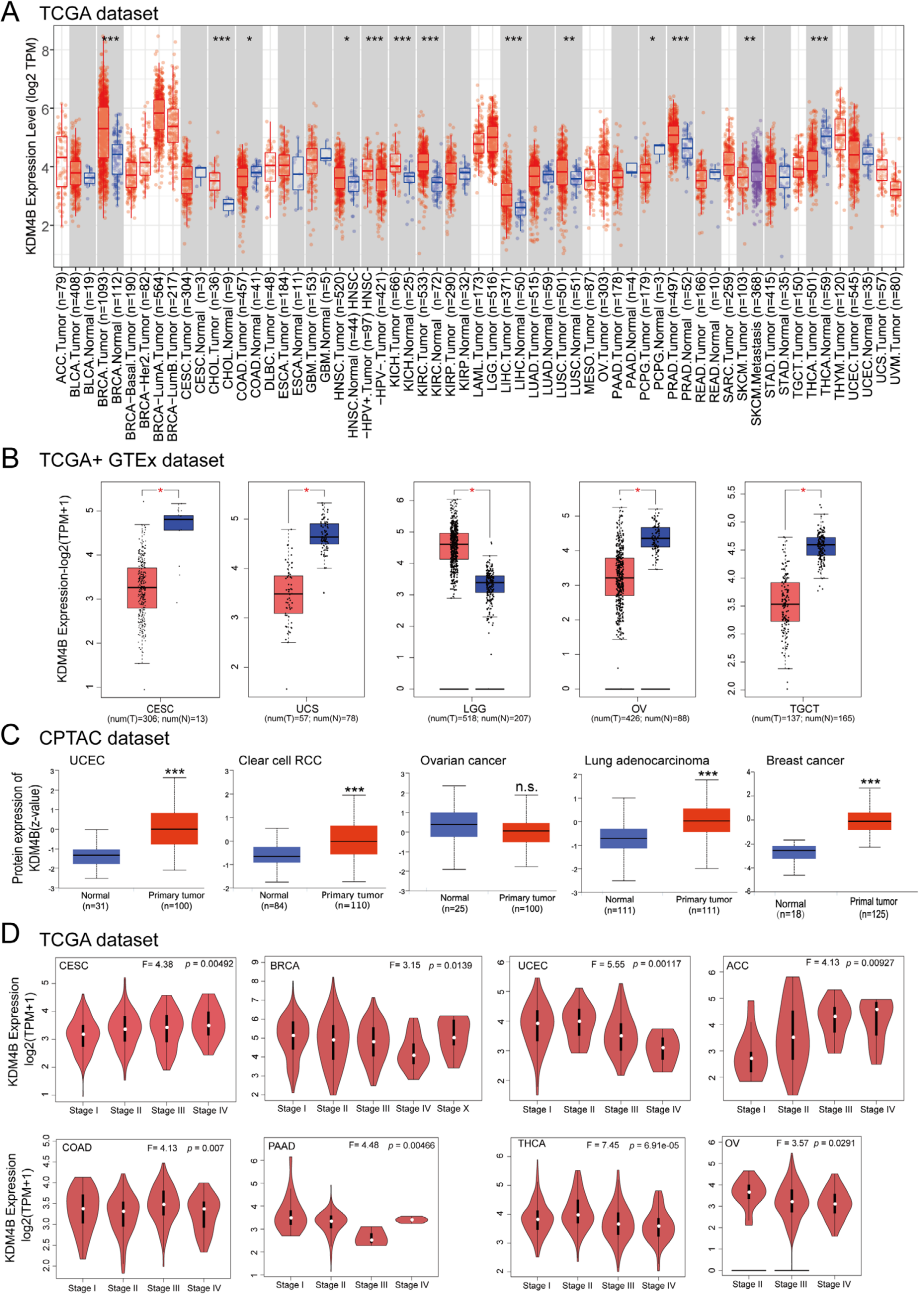
***KDM4B*** expression in different cancer types and pathological stages. (A) *KDM4B* expression levels in different cancer types or specific cancer subtypes were analyzed with Tumor IMmune Estimation Resource 2.0. * *p* < 0.05; ** *p* < 0.01; *** *p* < 0.001. (B) *KDM4B* expression in adrenocortical carcinoma (ACC), lymphoid neoplasm diffuse large B‐cell lymphoma, acute myeloid leukemia, lower‐grade brain glioma (brain lower grade glioma), OV (ovarian serous cystadenocarcinoma), sarcoma, testicular germ cell tumors, thymoma and uterine carcinosarcoma from the TCGA project were also analyzed along with their corresponding normal tissues from the GTEx database. Data exhibited as box plot. * *p* < 0.05. (C) KDM4B total protein levels were analyzed in normal tissue and primary tumor tissue of breast cancer, ovarian cancer, lung cancer, clear cell RCC and uterine corpus endometrial carcinoma (UCEC) from the Clinical Proteomic Tumor Analysis Consortium dataset. *** *p* < 0.001. (D) *KDM4B* expression levels were analyzed across the main pathological stages (Stages I–IV) of ACC, BRCA, colon adenocarcinoma, OV, cervical squamous cell carcinoma and endocervical adenocarcinoma, pancreatic adenocarcinoma, UCEC and thyroid carcinoma based on TCGA data. Log_2_ (transcripts per million + 1) was applied for log‐scale

Since we observed a common KDM4B regulatory mechanism among OVCAR, CRC, and RCC, we next sought to further evaluate the potential benefit of targeting the KDM4B pathway in different cancers. The expression status of *KDM4B* across various cancer types from The Cancer Genome Atlas (TCGA) was analyzed with the TIMER2.0 analysis tool (http://timer.cistrome.org/). As shown in Figure 6A, *KDM4B* is overexpressed in the tumor tissues of invasive breast carcinoma (BRCA), cholangiocarcinoma, head and neck squamous cell carcinoma (HNSC), chromophobe kidney cancer, clear cell RCC, hepatocellular carcinoma (LIHC), squamous cell lung carcinoma, prostate adenocarcinoma, and lower‐grade brain glioma (LGG), compared to their corresponding control tissues (Figure 6A, *p* < 0.05).

After including the normal tissue of the Genotype‐Tissue Expression (GTEx) dataset as controls, we further evaluated the expression difference of *KDM4B* between the normal tissues and tumor tissues of several more cancer types, where *KDM4B* expression was significantly higher in LGG tumors but lower in ovarian serous cystadenocarcinoma (OV), uterine carcinosarcoma (UCS), cervical squamous cell carcinoma and endocervical adenocarcinoma (CESC), and testicular germ cell tumor (TGCT) tissues, compared to their corresponding normal tissues (Figure 6B, *p *< 0.05). We did not observe a significant difference in other tumors, such as diffuse large B‐cell lymphoma, adrenocortical carcinoma (ACC), acute myeloid leukemia, sarcoma (SARC), or thymoma (Figure S2A). The results of the analysis with data in the Clinical Proteomic Tumor Analysis Consortium (CPTAC) dataset showed higher expression of KDM4B total protein in the primary tumor tissues of uterine corpus endometrial carcinoma (UCEC), ovarian cancer, breast cancer, clear cell RCC, and lung adenocarcinoma (LUAD) than in normal tissues (Figure 6C, *p *< 0.001).

We also used the “pathological stage plot” module of GEPIA2 (Gene Expression Profiling Interactive Analysis, version 2) to analyze the correlation between *KDM4B* expression and the pathological stages of cancers and found robust *KDM4B* expression in ACC, BRCA, colon adenocarcinoma, OV, pancreatic adenocarcinoma, thyroid carcinoma (THCA), CESC, and UCEC (Figure 6D, *p *< 0.05) but not other cancers (Figure S2B). These data demonstrate that *KDM4B* is overexpressed in multiple cancer tissues and is differentially expressed in different cancer stages, suggesting that KDM4B plays an important role in cancer progression, where the common KDM4B targets and pathways found in this study may also apply to other cancer types. Thus, further studying KDM4B regulatory mechanism in various cancer types may help discover targeted therapy that can benefit a broader spectrum of cancer patients.

### 2.7 High *KDM4B* expression correlates with improved or poor patient prognosis in a manner dependent on specific cancer types

**FIGURE 7:**
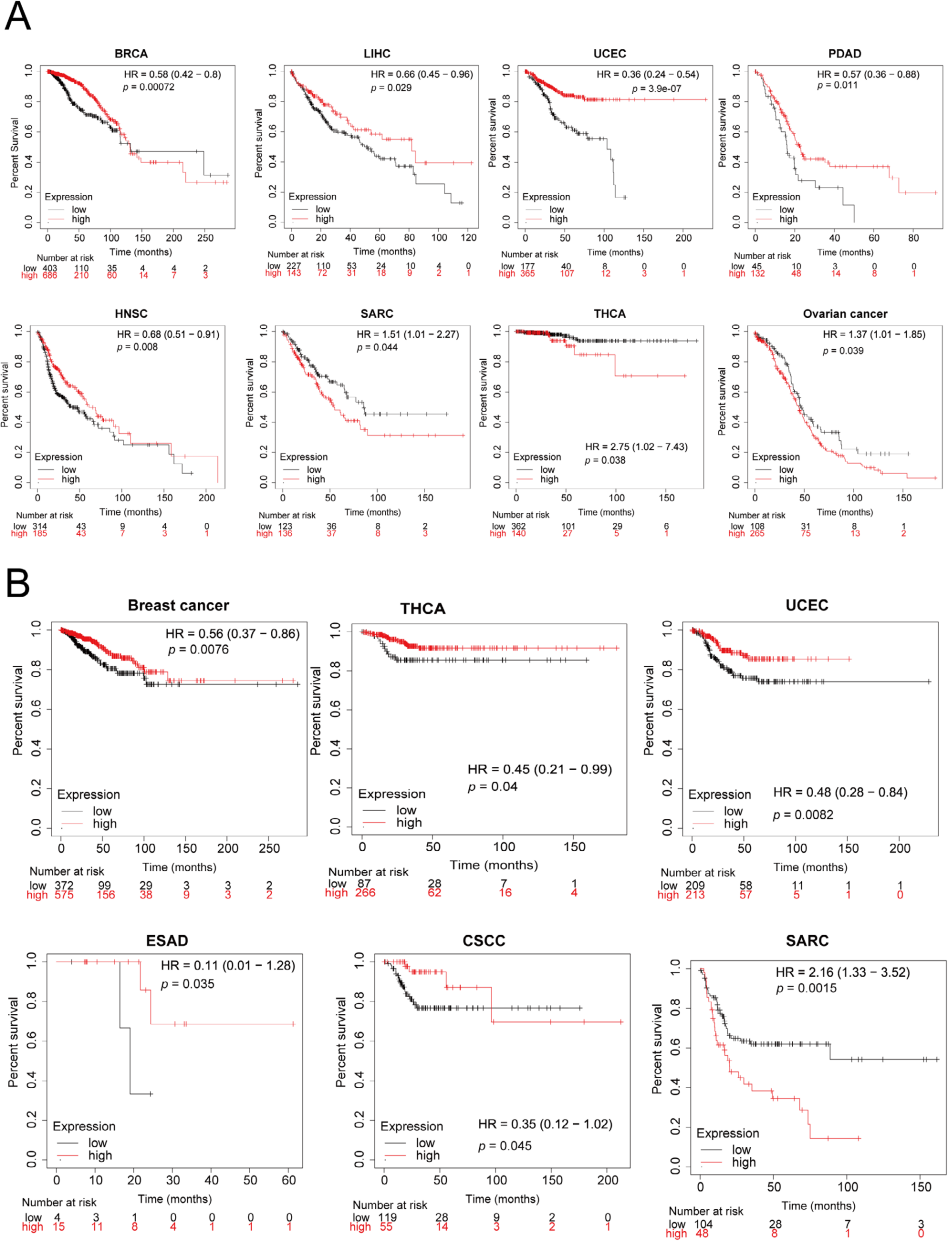
Association between *KDM4B* expression and the prognosis of patients with different tumors. (A) Kaplan–Meier plot analyzed with data from the pan‐cancer RNA‐seq project showing the correlation of *KDM4B* expression with patient overall survival, cancers with *p* < 0.05 were displayed. (B) Kaplan–Meier plot analyzed with data from the TCGA database showing the correlation of *KDM4B* expression with patient relapse‐free survival, cancers with *p* < 0.05 were displayed

We classified cancer cases into high *KDM4B* and low *KDM4B* expression groups and utilized the Kaplan–Meier plotter tool to investigate the correlation of *KDM4B* expression with the prognosis of patients with various cancers.^25^ As shown in Figure 7A, high *KDM4B* expression was coupled with a good prognosis of overall survival (OS) for patients with BRCA, LIHC, pancreatic ductal adenocarcinoma, HNSC, and UCEC within the pan‐cancer RNA‐seq project (Figure 7A, *p *< 0.05). Relapse‐free survival (RFS) analysis data revealed a correlation between high *KDM4B* expression and good patient prognosis of the TCGA cases with breast cancer, THCA, cervical squamous cell carcinoma, esophageal adenocarcinoma, and UCEC (Figure 7B, *p *< 0.05). Additionally, high *KDM4B* expression was linked to poor OS prognosis for SARC, THCA, ovarian cancer, and poor RFS prognosis for SARC (Figure 7, *p *< 0.05). These results revealed that *KDM4B* expression could be either positively or negatively correlated to cancer patient prognosis, suggesting KDM4B function in cancer cells is highly complex and distinct among different cancer types.

## 3 DISCUSSION

In this study, we have identified general, tissue‐specific, and oxygen‐specific KDM4B regulatory networks that may affect cancer progression. By using microarray analysis, we have identified 26 KDM4B‐dependent genes (16 genes were positively regulated by KDM4B, and 10 genes were negatively regulated by KDM4B) commonly shared by SKOV3ip.1, HCT116, and RCC4 cells, indicating a general regulation of KDM4B in different cancer types (Figure 1). Most of the commonly regulated genes have been reported to be associated with tumorigenesis and metastasis (Table S1). Two of the 16 positively regulated genes, *ERO1L* and *LOXL2*, are involved in vascular endothelial growth factor (VEGF) secretion and extracellular matrix (ECM) remodeling, respectively, suggesting KDM4B is essential for the general malignant phenotypes that are shared by multiple cancers.^26^, ^27^ Given the distinct phenotypes of ovarian, colon, and RCCs, investigation of these common target genes can help us understand better the general mechanism of cancer progression, which may help discover pan‐therapeutic methods simultaneously targeting multiple cancer types. Nonetheless, even though generally shared mechanisms may exist, cancer itself is a highly diverse disease,^28^ and different patients with the same type of cancer often have distinct pathological phenotype and therapeutic response.^29^ With the help of single‐cell sequencing technology, we now know that there are multiple distinct cellular populations with specific phenotypic, genetic, and epigenetic features within the same tumor.^29^ Therefore, studying the differentially regulated KDM4B targets in each cancer type and each patient may facilitate the design of precision therapies.

The hypoxic tumor microenvironment stimulates angiogenic mechanisms to recruit new blood vessels for oxygen restoration and induces the epithelial‐mesenchymal transition (EMT), ECM remodeling, and chemotaxis, which facilitates metastasis.^30^ Consistent with our previous report, KDM4B regulated different pathways in normoxia and hypoxia, indicating its multifunctional role in cancer progression^13^ (Figures 2A and 4A). In normoxia, it preferentially regulated proliferative and biosynthetic genes, maintaining cell functions to support tumor growth. In hypoxia, it switches to predominantly regulating inflammatory and migration‐related genes, possibly promoting invasion and metastasis. Sustaining proliferative signaling and activating invasion/metastasis are hallmarks of cancer.^31^ This dual function of KDM4B in cancer progression indicates its potential for serving as a cancer therapeutic target.

Our KEGG analyses discovered a COVID‐19‐associated pathway regulated by KDM4B in hypoxia (Figures 2C and 4C). Since many genes regulated by KDM4B in hypoxia were associated with the immune/inflammatory response, KDM4B‐regulated pathways may provide opportunities to better understand the progression of COVID‐19 infection. Approximately 10% or fewer hypoxia‐induced genes are regulated by KDM4B (Figures 2E and 4E). Interestingly, a large portion of KDM4B‐dependent genes is not induced by hypoxia, suggesting that KDM4B is important for maintaining gene expression levels during hypoxic stress, regulating genes independently of HIF.

KDM4B function has been studied in many cancer types (reviewed by Wilson et al.^15^). In our analysis of public cancer databases, we also found that it was expressed in high levels in many cancer types, with stage‐dependent expression changes (Figure 6). However, when analyzed with the Kaplan–Meier plotter tool, we found that high *KDM4B* did not always correlate with poor prognosis (Figure 7). Kaplan–Meier curves have improved outcomes for high KDM4B in breast cancer and uterine cancer. One possible reason for this phenotype may be that estrogen receptors (ERs) regulate KDM4B in ER+ breast cancer,^9^ which makes KDM4B correlated to a subtype of cancer that is more effectively treated with Tamoxifen and other anti‐estrogens. The same is true for uterine cancer, since antiprogestins, Tamoxifen, and aromatase inhibitors are also commonly used for its treatment, and estrogen responsiveness is a sign of a more differentiated phenotype.^32^ The tumor suppressor p53 regulates KDM4B,^33^ thus decreases in KDM4B expression may reflect the loss of p53 during disease progression as occurs in > 90% of high‐grade serous ovarian cancers.^34^ These data further support that KDM4B plays important and complicated roles in cancer progression.

Collectively, our study demonstrates a significant role of KDM4B in regulating tumorigenic gene expression, implying the therapeutic potential for KDM4B and its downstream targets.

## 4 MATERIALS AND METHODS

### 4.1 Culture conditions of cell lines

RCC4 and HCT116 cell lines were cultured in DMEM (Hyclone) with 10% FBS and 1% pen‐strep (P/S, Hyclone). SKOV3ip.1 cells were cultured as described previously.^13^ RCC4 and HCT116 cell lines were kindly provided by Amato Giaccia Lab (Stanford University). For short‐term *KDM4B* knockdown experiments, cells were transiently transfected with siRNA (Dharmacon siGenome Smartpool) specifically targeting *KDM4B* (siK4B, Thermo Fisher Scientific) using DharmaFECT 1 transfection reagent (Thermo Fisher Scientific) following the manufacturer's protocol. Scrambled siRNA (Dharmacon SiControl 2) was used as the control group (siCon, Thermo Fisher Scientific). For hypoxia treatment, cells were cultured for 16 h in Ruskinn InVivo300 glove‐box hypoxic incubators (Baker), where oxygen levels were set at either 2% or 0.5%.

### 4.2 QPCR

QPCR was conducted according to previously described methods.^13^ Briefly, total RNA was isolated with TRiReagent (Sigma‐Aldrich) from approximately 3 × 10^5^ cells following the manufacturer's protocol. One microgram of total RNA was reverse‐transcribed using the M‐MLV reverse transcriptase (Thermo Fisher Scientific) and 5 μM random primers (Thermo Fisher Scientific) following the manufacturer's protocol. For each QPCR reaction, roughly 0.625% of each diluted RT reaction product (2.5 μl in volume) was mixed with 2X SYBR green master mix (5 μl, Thermo Fisher Scientific) along with forward and reverse primers (50 nM, 0.4 μl) specific for the interested genes up to a total volume of 10 μl. Real‐time PCR signals were detected with an Applied Biosystems ViiA™ 7 Real‐Time PCR System (Thermo Fisher Scientific). Primers specific for 18S rRNA were used as an internal control. The online Roche Universal Probe Library design Tool (http://lifescience.roche.com/shop/en/us/overviews/brand/universal‐probe‐library) was used to design QPCR primers (Table 1). Melt curve analysis was used to confirm single amplicon formation.

**TABLE 1:** Primers used for QPCR

Gene	Forward (5′‐3′)	Reverse (5′‐3′)
*hsKDM4B*	ggactgacggcaacctctac	cgtcctcaaactccacctg
*hsCLDN12*	gctgttttggaactgtcaggta	ttccacacaggaaggaaagg
*hsLUZP6*	ggagacttggatgaggtgaaag	caccttctagtgtccggttga
*hsSEPT2*	tctgaagctgagaaccatgc	cataatgaaggtcctgggtcac
*hsMMD*	tctttttctatctcacaatgggatt	ctgaagtccatcggtgttgtt
*hsLOXL2*	tgacctgctgaacctcaatg	tggcacactcgtaattcttctg
*hsOSMR*	tgtctggagaattgtgagcttg	catgcagttttgataatggcttc
*hsPALLD*	aacaccagctgtcctgcttt	ggccttctttggaaatcctagt
*hsERO1L*	ggagacagcggcacagag	caatggtttcaacatcacaggt
*hsUSP6NL*	tgaggaggagctcccagat	ttcaatttccaggtgcttttg
*hsELAVL1*	cctcgtggatcagactacaggt	ctgggggtttatgaccattg
*hsBACE2*	tccatctacctgagagacgaga	tgggctgaatgtaaagctga
*hsTWSG1*	gtgagcaaatgcctcattca	cactccttacagcaggagcaa
*hsZAK*	tgacagagcagtccaacacc	gacatgacatctctgcactgttt
*hsBRCA2*	cctgatgcctgtacacctctt	gcaggccgagtactgttagc
*hsSKAP2*	tggagctttttctgatgagttgt	tctcctacccaccagccata
*hsCEP70*	gctgatcccagcctaacct	cccaacgagtctcatgtctg
*hsUCK2*	tccagatccccgtgtatgac	acgtctgcgggatagacagt
*hsMAP3K5*	cacgtgatgacttaaaatgcttg	agtcaatgatagccttccacagt
*hsMAP4K3*	agctttggatttgcatggag	ttctgacagaggtccagttacg
*hsMAP4K4*	caggacaagctcactgctaatg	tctggggtctataaaaggtgtaaag
*hsMEF2Av1*	tgatgcggaatcataaaatcg	tggaactgtgacagacattgaa
*hsMEF2Av2*	tgaagatagtgattttattttcaaacg	gtgacagacattgaaaagttctgag
*hsTRAF1a*	ctgtgcaggctgtctctctg	cggcttcctgggcttatag
*hsTNFSF14*	agcgaaggtctcacgaggt	cggtcaagctggagttgg
*hsLPP*	cactgcattgaggacttcca	caaagccacaatacggacag
*hsITGB8*	gcattatgtcgaccaaacttca	gcaacccaatcaagaatgtaact
*hsFGFRL1*	cagcctgagcgtcaactaca	ctctccttccctgggctaat
*hsITGB5*	ggagtttgcaaagtttcagagc	tgtgcgtggagataggcttt
*hsPDGFB*	ctggcatgcaagtgtgagac	cgaatggtcacccgagttt
*hsMYD88*	ttctcggaaagcgaaagc	attgtctgccagcgcttc
*hsTLR3*	agagttgtcatcgaatcaaattaaag	aatcttccaattgcgtgaaaa
*hsTLR5*	ctgtccgaacctggagaca	tcctgagactataggaatctcatcac
*hsIL8*	agacagcagagcacacaagc	atggttccttccggtggt
*hsSMAD3*	tagctcccggtagaggatca	aaggctgggaaaagaagagg
*hsIRAK2*	aacttgtggacctcctgtgc	ccggtttccagttcaggat
*hsIL6*	gatgagtacaaaagtcctgatcca	ctgcagccactggttctgt
*hsIL6ST*	aggaccaaagatgcctcaac	gaatgaagatcgggtggatg
*hsIL1β*	ctgtcctgcgtgttgaaaga	ttgggtaatttttgggatctaca
*hsIL1R1*	ccaagaagaatatgaaagtgttactca	ttcttcacgttccttgcattt
*hsIL1R2*	cagaaagagcttctgaaggaagac	acacgggaagtggaggact
*hsFYN*	agattgctgacttcggattg	cagacttgattgtgaacctc
*18S rRNA*	gcccgaagcgtttactttga	tccattattcctagctgcggtatc

### 4.3 Microarray gene expression analysis

All three cell lines were transfected with siK4B (Thermo Fisher Scientific) or siCon (Thermo Fisher Scientific) for 32 h (transfection was repeated after the first 24 h) before culturing in 21% O_2_ (all three cell lines) or 0.5% O_2_ (SKOV3ip.1 and HCT116 cell lines) for 16 more h. Each RNA sample (100 ng) was profiled using the GeneChip® Human Exon 1.0 ST Arrays (Affymetrix, Thermo Fisher Scientific). Normalization and differential gene expression analysis was performed in the Partek Genomic suite (v 6.5, Partek Inc.). These exon arrays were RMA (Robust Multi‐Array Average)‐background‐corrected, quantile‐normalized, and gene‐level‐summarized with the Median Polish algorithm.^35^ The resulting expression values (log_2_‐transformed signal intensities) were used in a two‐way analysis of variance model to calculate differential expression. *p*‐values were corrected for multiple hypothesis testing using the Benjamini and Hochberg method.^36^ The analysis was done on samples obtained from biological triplicates. Genes with ≥1.4‐fold significant up‐ or down‐regulation by si*KDM4B* knockdown (*p *< 0.05), compared to the siControl cells, were considered as potential KDM4B downstream targets. Genes with ≥1.4‐fold significant up‐regulation when comparing siControl in hypoxia (0.5% O_2_) to siControl in normoxia (21% O_2_) were considered as hypoxia‐induced genes.

### 4.4 Pathway analyses

Venny 2.1 online analysis tool (by Juan Carlos Oliveros Bioinfo GP, CNB‐CSIC, https://bioinfogp.cnb.csic.es/tools/venny/) was employed to identify overlapping expression among the three cell lines, as well as between the hypoxia‐induced genes and KDM4B targets. Hypergeometric distribution was used to calculate the statistical significance of overlap regions between the cell lines and oxygen conditions.^37^ GO biological process and KEGG pathway enrichment analyses were performed using enrichGO and enrichKEGG from the “clusterProfiler” R package. Results were visualized with R version 4.0.2. Functional pathways influenced by KDM4B were also identified using DAVID Functional Annotation Tool (DAVID Bioinformatics Resources 6.8, NIAID/NIH, https://david.ncifcrf.gov/summary.jsp). The PALs were calculated through the Oncobox library (https://open.oncobox.com).^24^ Microarray data were deposited under accession numbers GSE166991 and GSE167025 in the NCBI's Gene Expression Omnibus.^38^

### 4.5 *KDM4B* gene expression analysis in clinical samples

*KDM4B* expression across clinical cancer samples was analyzed with the Tumor IMmune Estimation Resource (TIMER2.0, version 2, http://timer.cistrome.org/) online software. Briefly, “*KDM4B*” was entered in the “Gene_DE” module of the TIMER2.0 website, and the difference of *KDM4B* expression between cancerous tissues and their adjacent normal tissues was analyzed for different cancers or specific cancer subtypes from the TCGA database.

For cancers with no or limited normal tissue samples, such as CESC, OV, UCS, LGG, and TGCT, we used the “Expression analysis‐Box Plots” module of the GEPIA2 online server (http://gepia2.cancer‐pku.cn/#analysis) to generate box plots comparing *KDM4B* expression between cancer tissues and their respective normal tissues from the GTEx database, with *p*‐value cutoff set as 0.01, log_2_(fold change) cutoff set as 1, as well as matched TCGA normal and GTEx data.

The violin plots of the *KDM4B* expression were obtained from TCGA tumors in different pathological stages (Stages I– IV) via the “Pathological Stage Plot” module of GEPIA2. We applied the log_2_ [transcripts per million +1]‐ transformed expression data for the violin or box plots. The UALCAN portal (http://ualcan.path.uab.edu/analysis‐prot.html), an interactive online server for analysis of cancer Omics data, was used to analyze *KDM4B* expression of the CPTAC dataset. Here, we explored the total KDM4B protein expression level between primary cancer tissues and their corresponding normal tissues by entering “*KDM4B*.” The available datasets of five different cancers were selected, namely, OVCA, RCC, breast cancer, UCEC, and LUAD.

### 4.6 Survival analysis

Cases of each cancer type were classified into *KDM4B* high and *KDM4B* low groups according to the optimal cutoff value suggested by the Kaplan–Meier Plotter browser (https://kmplot.com). The Kaplan–Meier Plotter browser is an online database (sources include GEO, EGA, and TCGA) capable of assessing the effect of 54,000 genes (mRNA, miRNA, protein) on survival in 21 cancer types. The survival differences between *KDM4B* high and *KDM4B* low groups were visualized by generating Kaplan–Meier survival plots. The log‐rank *p*‐values and hazard ratios with 95% confidence intervals were also computed.

## CONFLICT OF INTEREST

The authors declare no conflict of interest.

## ETHICS STATEMENT

Clinical data was collected from approved online databases including TCGA, GTEx and CAPTC datasets, ethics issues are not applicable.

## AUTHOR CONTRIBUTIONS

Research design: Lei Qiu, Yang Meng, Adam Krieg, Junhong Han. Original draft writing and editing: Lei Qiu, Yang Meng, Adam Krieg, Junhong Han. Data analyses: Lei Qiu, Yang Meng, Lingli Wang, Sumedha Gunewardena, Sicheng Liu, Adam Krieg. Figure creating: Lei Qiu, Yang Meng, Lingli Wang. Experiment: Lei Qiu.

## Supporting information

Supporting Information

## Data Availability

Microarray data was deposited under accession numbers GSE166991 and GSE167025 in the NCBI's Gene Expression Omnibus.
