# Supplementary material for: Histone lysine demethylase 4B regulates general and unique gene expression signatures in hypoxic cancer cells
Source: MedComm (2020). 2021 Aug 27;2(3):414–29. doi: 10.1002/mco2.85 (PMC8554665; doi:10.1002/mco2.85)
Supplement: Supplementary file 1 — Supporting Information [file MCO2-2-414-s001.docx]

**KDM4B Regulates Shared and Tissue-specific Pathways in Three Different Cancers Dependent or Independent of HIF signaling**

Lei Qiu^1,2,3*^, Yang Meng^1*^, Lingli Wang^1^, Yan Hong^2^, Sumedha Gunewardena^4^, Sicheng Liu^1^, Junhong Han^1#^, Adam J. Krieg^2,3,5#^

^1^Research Laboratory of Cancer Epigenetics and Genomics, Department of General Surgery, Frontiers Science Center for Disease-related Molecular Network, and Cancer Center, West China Hospital, Sichuan University, Chengdu, China; ^2^Department of Obstetrics and Gynecology, University of Kansas Medical Center, Kansas City, KS, USA; ^3^Department of Pathology and Laboratory Medicine, University of Kansas Medical Center, Kansas City, KS, USA; ^4^Department of Molecular and Integrative Physiology, University of Kansas Medical Center, Kansas City, KS, USA; ^5^Department of Obstetrics and Gynecology, Oregon Health and Science University, Division of Reproductive and Developmental Sciences, Oregon National Primate Research Center, Beaverton, OR, USA.

**Correspondence:**

^#^Adam J. Krieg, Department of Obstetrics and Gynecology, Oregon Health and Science University, Division of Reproductive and Developmental Sciences, Oregon National Primate Research Center, 505 NW 185th Avenue, Mail Stop L584, Beaverton, OR 97006, USA; E-mail: [kriega@ohsu.edu](mailto:kriega@ohsu.edu).

^#^Junhong Han, Research Laboratory of Cancer Epigenetics and Genomics, Department of General Surgery, Frontiers Science Center for Disease-related Molecular Network, and Cancer Center, West China Hospital, Sichuan University, Chengdu 610041, China; Telephone (86) 28-61528677; E-mail: [hjunhong@scu.edu.cn](mailto:hjunhong@scu.edu.cn).

*These authors contributed equally to this work.

**Supplemental Figure Legends**

**Figure S1. GO biological process analysis.** GO analysis of biological process using the 133+315 genes from Figure 1B overlapped in SKOV3ip.1 and RCC4 cells, excluding the 26 genes common in all three cell lines.

**Figure S2. Expression level of the KDM4B gene in different tumors and pathological stages. A,** The expression statuses of the KDM4B gene in ESCA (Esophageal carcinoma), DLBC (Lymphoid Neoplasm Diffuse Large B-cell Lymphoma), COAD (Colon adenocarcinoma), CHOL (Cholangio carcinoma), BLCA (Bladder Urothelial Carcinoma), ACC (Adrenocortical carcinoma), THYM (Thymoma), SARC (Sarcoma), LAML (Acute Myeloid Leukemiav), KIRP (Kidney renal papillary cell carcinoma), KIRC (Kidney renal clear cell carcinoma), KICH (Kidney Chromophobe), HNSC (Head and Neck squamous cell carcinoma), GBM (Glioblastoma multiforme) in TCGA project were compared with the corresponding normal tissues of the GTEx databases. **B,** Expression levels of the KDM4B gene by different pathological stages of BLCA, CHOL, DLBC, ESCA; KIRC, KIRP, LIHC (Liver hepatocellular carcinoma), STAD (Stomach adenocarcinoma), HNSC, READ (Rectum adenocarcinoma), LUAD (Lung adenocarcinoma), TGCT (Testicular Germ Cell Tumors), UCS (Uterine Carcinosarcoma).

**Figure S3. Correlation between KDM4B expression** **and patient overall survival (OS).** The correlation was analyzed within the pan-cancer RNA-seq project; cancer types shown here displayed no biological significance.

**Figure S4. correlation between KDM4B expression and patient relapse-free survival (RFS).** The correlation between KDM4B expression and patient relapse-free survival (RFS) were analyzed with cases from TCGA database; cases shown here displayed no biological significance.

**Figure S1**


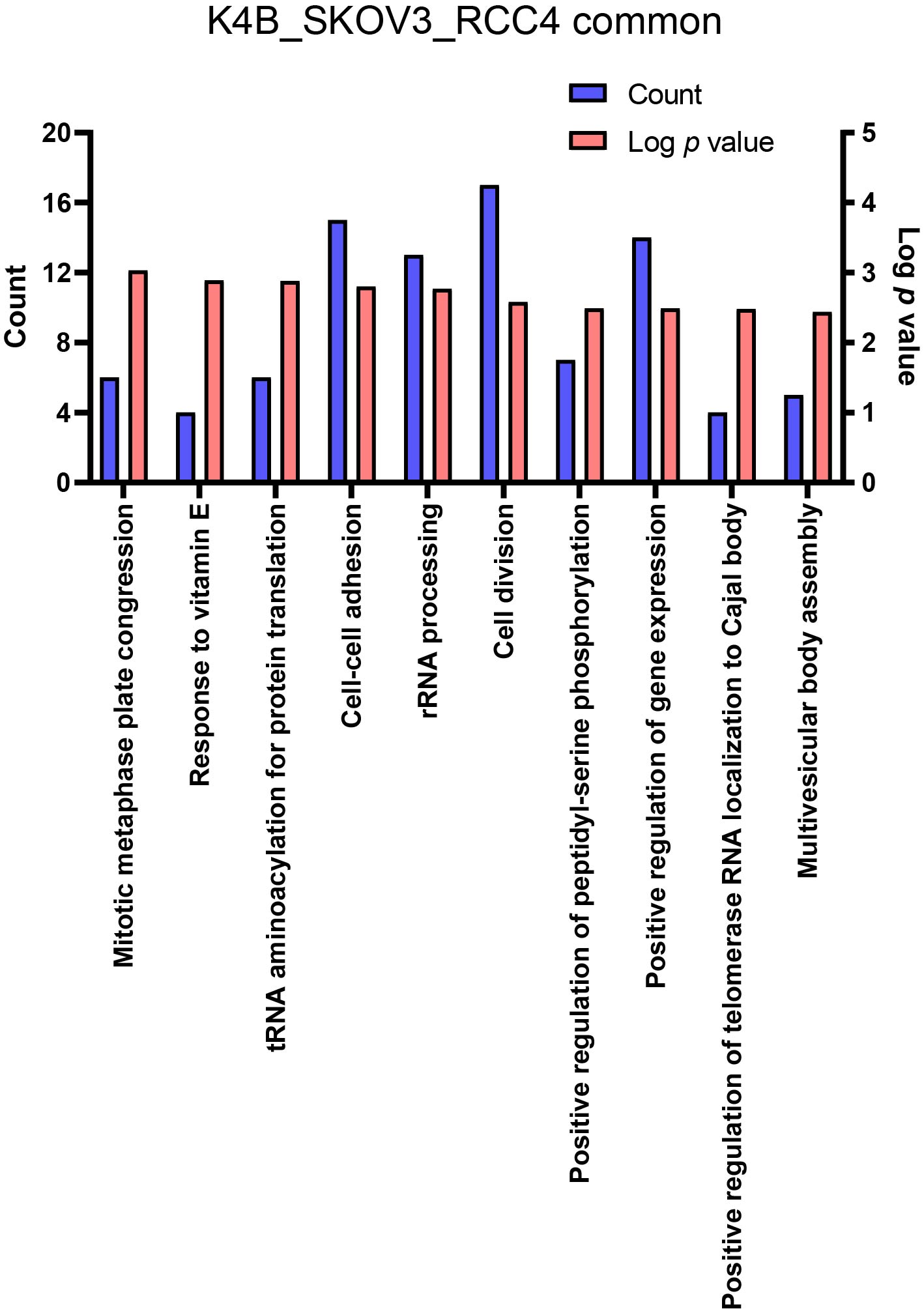


**Figure S2**


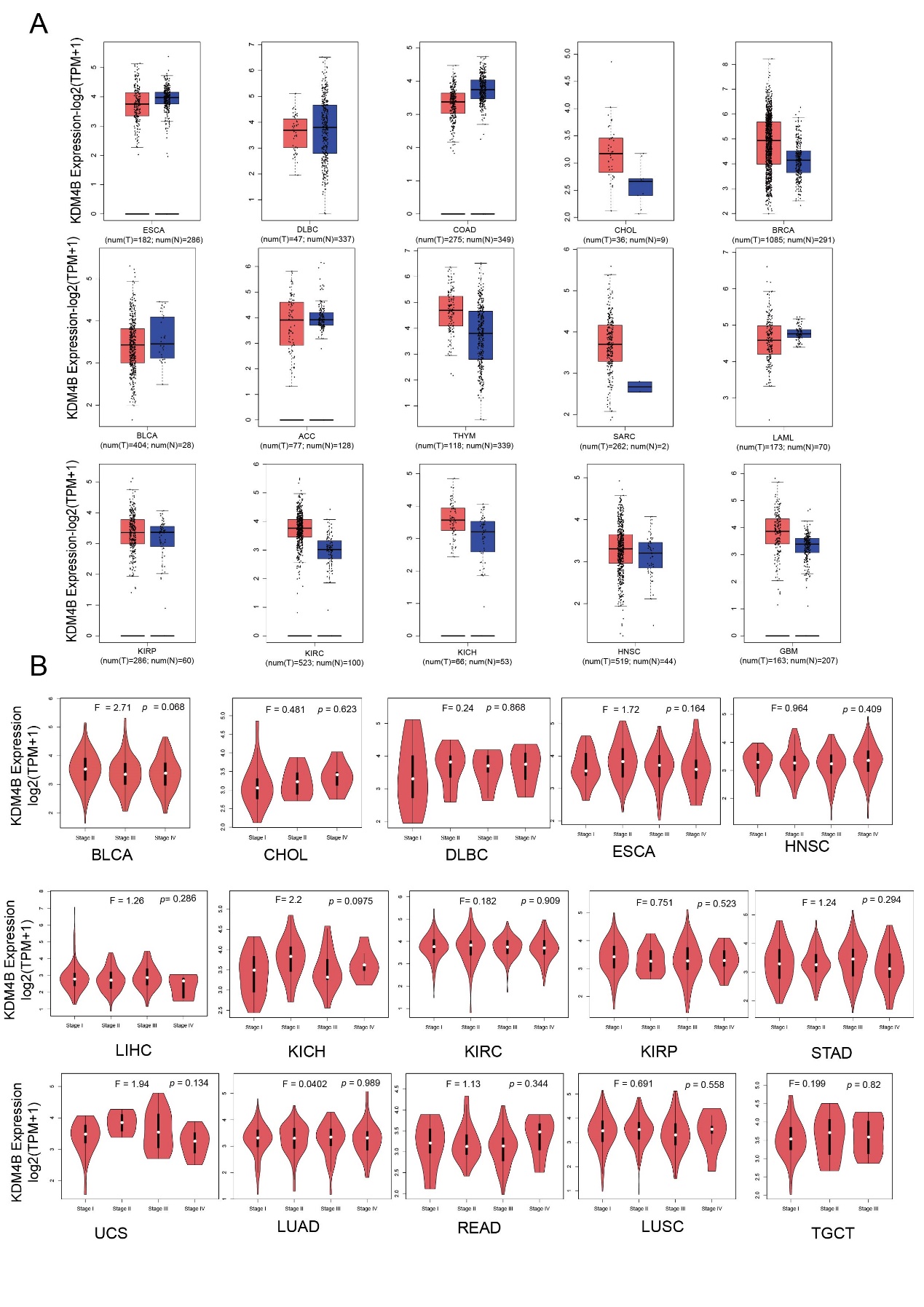


**Figure S3**


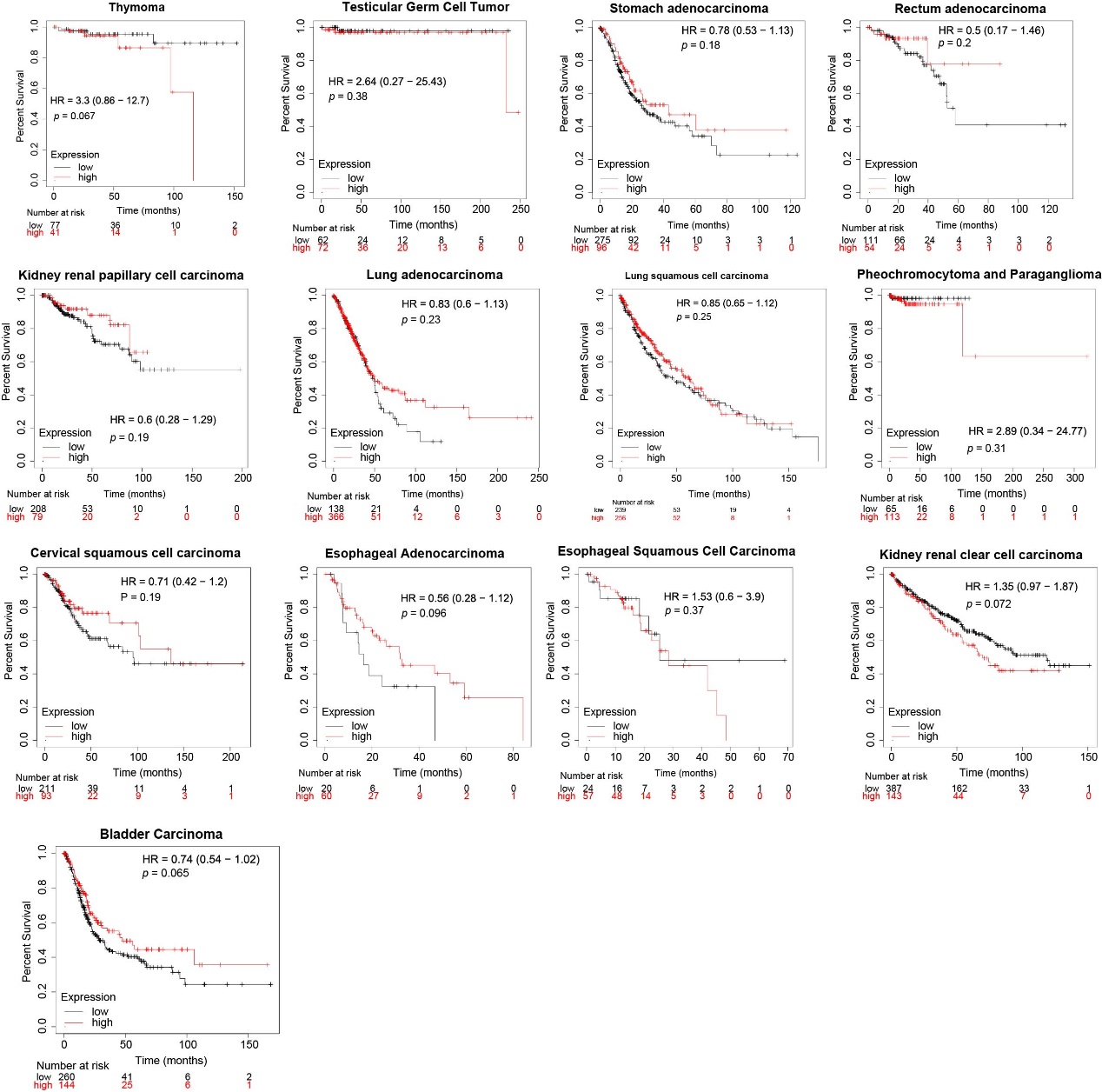


**Figure S4**


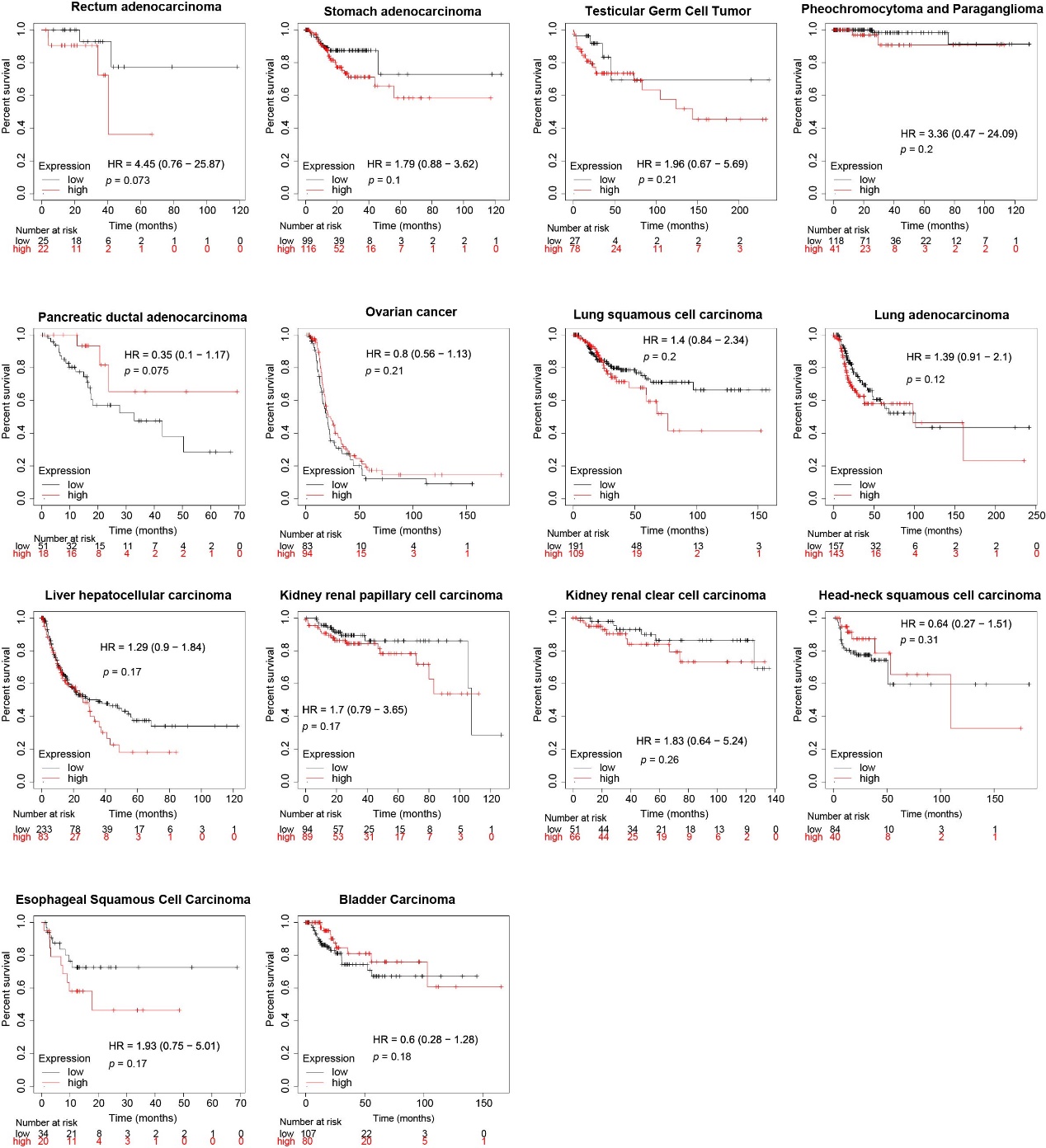


**Table S1. Description of the 26 common genes regulated by KDM4B in SKOV3ip.1, HCT116 and RCC4 cell lines.**

| **Gene Symbol** | **Fold Change** | | | **Gene Description** | **Cellular Process** | **Related Disease** |
| --- | --- | --- | --- | --- | --- | --- |
|  | **SKOV3ip.1** | **HCT116** | **RCC4** |  |  |  |
| ***CLDN12*** | -2.24 | -1.52 | -1.7 | Claudin 12 | Tumorigenesis[^1^](#_ENREF_1), chemoresistance[^2^](#_ENREF_2), metastasis[^3^](#_ENREF_3) | Colorectal cancer[^1^](#_ENREF_1), breast cancer[^2^](#_ENREF_2), lung squamous cell crcinoma[^3^](#_ENREF_3), osteosarcoma[^4^](#_ENREF_4) |
| ***LUZP6*** | -1.44 | -1.53 | -1.49 | Myotrophin; leucine zipper protein 6 | Myeloproliferative disease-associated antigen[^5^](#_ENREF_5) |  |
| ***SEPT2*** | -1.97 | -1.46 | -2.24 | Septin 2 | Cell proliferation[^6^](#_ENREF_6), carcinogenesis[^7^](#_ENREF_7), migration[^8^](#_ENREF_8) | Colorectal cancer[^9^](#_ENREF_9), breast cancer [^8^](#_ENREF_8), prostate cancer[^10^](#_ENREF_10), hepatocellular carcinoma[^6^](#_ENREF_6), biliary tract cancer[^11^](#_ENREF_11), leukemia[^12^](#_ENREF_12), glioblastoma[^13^](#_ENREF_13) |
| ***MMD*** | -4.4 | -1.63 | -5.49 | Monocyte to macrophage differentiation-associated | Tumor growth[^14^](#_ENREF_14), metastasis/EMT[^15^](#_ENREF_15), mediate Ras signaling in the Golgi apparatus[^16^](#_ENREF_16), monocyte-to-macrophage differentiation and phatogenesis[^17^](#_ENREF_17) | Lung and breast cancer [^15^](#_ENREF_15), non-small cell lung cancer[^14^](#_ENREF_14), rheumatoid arthritis[^17^](#_ENREF_17) |
| ***LOXL2*** | -3.18 | -1.62 | -3.28 | Lysyl oxidase-like 2 | Metastasis/EMT[^18^](#_ENREF_18), tumor microenvironment[^19^](#_ENREF_19), radiosensitivity[^20^](#_ENREF_20) | Cervical carcinoma[^18^](#_ENREF_18), prostate cancer[^19^](#_ENREF_19)^,^ [^20^](#_ENREF_20), breast cancer[^21^](#_ENREF_21), pancreatic cancer[^22^](#_ENREF_22), colorectal cancer[^23^](#_ENREF_23), Hepatocellular carcinoma[^24^](#_ENREF_24), osteosarcoma[^25^](#_ENREF_25), gastric cancer[^26^](#_ENREF_26), cervical cancer[^27^](#_ENREF_27), alveolar rhabdomyosarcoma[^28^](#_ENREF_28), head and neck squamous cell carcinoma[^29^](#_ENREF_29), intrahepatic cholangiocarcinoma[^30^](#_ENREF_30) |
| ***OSMR*** | -1.61 | -1.52 | -1.73 | Oncostatin M receptor | Angiogenesis and invasion[^31^](#_ENREF_31), radiosensitivity[^32^](#_ENREF_32), growth and metastasis[^33^](#_ENREF_33), cancer local immune response[^34^](#_ENREF_34), chemoresistance[^35^](#_ENREF_35) | Glioblastoma[^32^](#_ENREF_32), colorectal cancer[^36^](#_ENREF_36), bladder cancer[^37^](#_ENREF_37), gastric cancer[^33^](#_ENREF_33), pancreatic ductal adenocarcinoma[^38^](#_ENREF_38), squamous cell carcinoma[^39^](#_ENREF_39), medullary thyroid carcinoma[^40^](#_ENREF_40), non-small cell lung cancer[^35^](#_ENREF_35), breast cancer[^41^](#_ENREF_41), leukemia[^42^](#_ENREF_42), Ewing sarcoma[^43^](#_ENREF_43), osterosarcoma[^31^](#_ENREF_31), ovarian carcinoma [^44^](#_ENREF_44) |
| ***PALLD*** | -1.47 | -1.58 | -1.78 | Palladin, cytoskeletal associated protein | Invasion/metastasis[^45^](#_ENREF_45), anti-migration[^46^](#_ENREF_46), phagocytosis[^47^](#_ENREF_47) | Pancreatic cancer[^45^](#_ENREF_45), breast cancer[^46^](#_ENREF_46), colorectal cancer[^48^](#_ENREF_48), renal cell carcinoma[^49^](#_ENREF_49) |
| ***ERO1L*** | -3.38 | -1.43 | -2.85 | ERO1-like (S. cerevisiae) | Disulphide bond formation in ER[^50^](#_ENREF_50), cancer progression[^51^](#_ENREF_51), metastasis[^52^](#_ENREF_52), cell cycle[^53^](#_ENREF_53), tumorigenesis[^54^](#_ENREF_54) | Gastric cancer [^55^](#_ENREF_55), pancreatic cancer[^51^](#_ENREF_51), lung cancer[^52^](#_ENREF_52)^,^ [^53^](#_ENREF_53), oral squamous cell carcinoma[^54^](#_ENREF_54), multiple myeloma[^56^](#_ENREF_56) |
| ***USP6NL*** | -1.48 | -1.49 | -1.82 | USP6 N-terminal like | cell proliferation[^57^](#_ENREF_57), cancer development[^58^](#_ENREF_58) | Colorectal cancer[^57^](#_ENREF_57), breast cancer[^58^](#_ENREF_58), Alzheimer's disease[^59^](#_ENREF_59) |
| ***ELAV1*** | -1.6 | -1.45 | -1.63 | Embryonic lethal, abnormal vision, Drosophila-like 1 (Hu antigen R) | Regulation of RNA stability[^60^](#_ENREF_60), apoptosis[^61^](#_ENREF_61), DNA damage response[^62^](#_ENREF_62) | Urinary tumors[^63^](#_ENREF_63), breast cancer [^60^](#_ENREF_60) |
| ***BACE2*** | -1.81 | -1.4 | -1.8 | Beta-site APP-cleaving enzyme 2 | Cleaves Amyloid-beta-precursor protein[^64^](#_ENREF_64) | Alzhermer's disease[^65^](#_ENREF_65), diabetes [^66^](#_ENREF_66) |
| ***TWSG1*** | -1.94 | -1.45 | -2.39 | Twisted gastrulation homolog 1 (Drosophila) | Tumor suppressor[^67^](#_ENREF_67), cell motility and proliferation[^68^](#_ENREF_68) | Gastric cancer[^67^](#_ENREF_67), papillary thyroid cancer[^68^](#_ENREF_68) |
| ***ZAK*** | -1.65 | -1.54 | -1.64 | Sterile alpha motif and leucine zipper containing kinase AZK | Cardiac compensation[^69^](#_ENREF_69), cellular hypertrophy[^70^](#_ENREF_70), ECM remodeling[^71^](#_ENREF_71), TGF-β signaling[^72^](#_ENREF_72), tumor cell invasion[^73^](#_ENREF_73), radiosensitivity[^74^](#_ENREF_74), pro-apoptotic signaling p38/JNK[^75^](#_ENREF_75) | Lung and breast cancer[^72^](#_ENREF_72) |
| ***ZDHHC2*** | -1.53 | -1.49 | -2.06 | Zinc finger, DHHC-type containing 2 | Metastasis[^76-78^](#_ENREF_76) | Hepatocellular carcinoma[^77^](#_ENREF_77), gastric adenocarcinoma[^78^](#_ENREF_78), nasopharyngeal carcinoma[^76^](#_ENREF_76), renal clear cell carcinoma[^79^](#_ENREF_79) |
| ***DENND5A*** | -1.62 | -1.4 | -2.6 | DENN/MADD domain containing 5A | MDCK cystogenesis[^80^](#_ENREF_80), tumor suppressor[^81^](#_ENREF_81) | Colorectal cancer[^81^](#_ENREF_81) |
| ***DAZAP2*** | -2.5 | -1.52 | -3.73 | DAZ associated protein 2 | TCF4 binding factor and coactivator[^82^](#_ENREF_82), Posterior neural patterning[^83^](#_ENREF_83), ectoderml differentiation[^84^](#_ENREF_84) | Multiple myeloma[^85^](#_ENREF_85) |
| ***STARD7*** | 2.04 | 2.13 | 1.52 | StAR Related Lipid Transfer Domain Containing 7 | Wnt/β-catenin signaling pathway[^86^](#_ENREF_86), cell migration, proliferation, and differentiation[^87^](#_ENREF_87) | Gestational trophoblast tumor[^88^](#_ENREF_88), choriocarcinoma[^87^](#_ENREF_87) |
| ***GEMIN5*** | 1.97 | 1.71 | 2.08 | Gem Nuclear Organelle Associated Protein 5 | Alternative mRNA splicing patterns and tumor cell motility[^89^](#_ENREF_89) | Breast cancer[^89^](#_ENREF_89) |
| ***CPA4*** | 1.75 | 1.47 | 2.15 | Carboxypeptidase A4 | Proliferation[^90^](#_ENREF_90)^,^ [^91^](#_ENREF_91), stem cell characteristics[^91^](#_ENREF_91), EMT[^92^](#_ENREF_92) | Hepatocellular carcinoma[^91^](#_ENREF_91), colorectal cancer[^90^](#_ENREF_90), non-small-cell lung cancer[^93^](#_ENREF_93), Breast Cancer[^94^](#_ENREF_94), pancreatic cancer[^92^](#_ENREF_92) |
| ***FKBP14*** | 2.20 | 1.57 | 2.58 | FKBP Prolyl Isomerase 14 | Carcinogenesis[^95^](#_ENREF_95), proliferation, adhesion and invasion[^96^](#_ENREF_96) | Osteosarcoma[^95^](#_ENREF_95), gastric cancer[^96^](#_ENREF_96), Ovarian Cancer[^97^](#_ENREF_97) |
| ***CARNMT1*** | 2.47 | 1.49 | 3.07 | Carnosine N-Methyltransferase 1 |  |  |
| ***MPZL3*** | 2.65 | 1.51 | 1.65 | Myelin Protein Zero Like 3 | Metabolism[^98^](#_ENREF_98), adhesive and immune functions[^99^](#_ENREF_99), Epidermal Differentiation[^100^](#_ENREF_100), hair cycle clock[^101^](#_ENREF_101) | Obesity[^98^](#_ENREF_98), immune-related hereditary hair loss[^99^](#_ENREF_99) |
| ***DYRK2*** | 2.10 | 1.47 | 2.03 | Dual Specificity Tyrosine Phosphorylation Regulated Kinase 2 | Apoptotic induction[^102^](#_ENREF_102), suppresses carcinogenesis[^103^](#_ENREF_103), suppresses cell proliferation[^104^](#_ENREF_104), controls EMT[^105^](#_ENREF_105), suppresses migration and invasion[^106^](#_ENREF_106) | liver cancer[^102^](#_ENREF_102), colorectal cancer[^103^](#_ENREF_103), Non-Hodgkin's Lymphoma[^104^](#_ENREF_104), breast cancer[^105^](#_ENREF_105), colorectal cancer[^106^](#_ENREF_106) |
| ***KCTD10*** | 1.81 | 1.66 | 1.77 | Potassium Channel Tetramerization Domain Containing 10 | K27-polyubiquitination of EIF3D[^107^](#_ENREF_107), Rac1 activation through RhoB degradation[^108^](#_ENREF_108) | Gastrointestinal stromal tumor[^109^](#_ENREF_109), hepatocellular carcinoma[^107^](#_ENREF_107), breast cancer[^108^](#_ENREF_108) |
| ***RNF6*** | 1.63 | 1.49 | 1.44 | Ring Finger Protein 6 | Cell growth[^110^](#_ENREF_110)^,^ [^111^](#_ENREF_111), Activating the Wnt/β-Catenin Pathway via Ubiquitination of TLE3[^112^](#_ENREF_112) | Gastric cancer[^110^](#_ENREF_110), breast cancer[^111^](#_ENREF_111), colorectal cancer[^112^](#_ENREF_112) |
| ***HCCS*** | 2.08 | 1.57 | 1.95 | Holocytochrome C Synthase |  |  |

**References**

1. Grone J, Weber B, Staub E, et al. Differential expression of genes encoding tight junction proteins in colorectal cancer: frequent dysregulation of claudin-1, -8 and -12. *Int J Colorectal Dis*. 2007;22(6):651-9.

2. Du Y, Zhou L, Lin Y, Yin K, Yin W, Lu J. Polymorphisms in microRNA let-7 binding sites of the HIF1AN and CLDN12 genes can predict pathologic complete response to taxane- and platinum-based neoadjuvant chemotherapy in breast cancer. *Ann Transl Med*. 2019;7(7):138.

3. Sun L, Feng L, Cui J. Increased expression of claudin-12 promotes the metastatic phenotype of human bronchial epithelial cells and is associated with poor prognosis in lung squamous cell carcinoma. *Exp Ther Med*. 2019;17(1):165-174.

4. Tian X, He Y, Han Z, Su H, Chu C. The Cytoplasmic Expression Of CLDN12 Predicts An Unfavorable Prognosis And Promotes Proliferation And Migration Of Osteosarcoma. *Cancer Manag Res*. 2019;11:9339-9351.

5. Xiong Z, Liu E, Yan Y, et al. An unconventional antigen translated by a novel internal ribosome entry site elicits antitumor humoral immune reactions. *J Immunol*. 2006;177(7):4907-16.

6. Yu W, Ding X, Chen F, et al. The phosphorylation of SEPT2 on Ser218 by casein kinase 2 is important to hepatoma carcinoma cell proliferation. *Mol Cell Biochem*. 2009;325(1-2):61-7.

7. Liu M, Shen S, Chen F, Yu W, Yu L. Linking the septin expression with carcinogenesis. *Mol Biol Rep*. 2010;37(7):3601-8.

8. Cai B, Wang X, Bu Q, et al. LncRNA AFAP1-AS1 Knockdown Represses Cell Proliferation, Migration, and Induced Apoptosis in Breast Cancer by Downregulating SEPT2 Via Sponging miR-497-5p. *Cancer Biother Radiopharm*. 2020;

9. Zhang W, Liao K, Liu D. MicroRNA7445p is downregulated in colorectal cancer and targets SEPT2 to suppress the malignant phenotype. *Mol Med Rep*. 2021;23(1)

10. Xing Z, Li S, Liu Z, Zhang C, Meng M, Bai Z. The long non-coding RNA LINC00473 contributes to cell proliferation via JAK-STAT3 signaling pathway by regulating miR-195-5p/SEPT2 axis in prostate cancer. *Biosci Rep*. 2020;40(9)

11. Yu J, Zhang W, Tang H, et al. Septin 2 accelerates the progression of biliary tract cancer and is negatively regulated by mir-140-5p. *Gene*. 2016;589(1):20-26.

12. Cerveira N, Correia C, Bizarro S, et al. SEPT2 is a new fusion partner of MLL in acute myeloid leukemia with t(2;11)(q37;q23). *Oncogene*. 2006;25(45):6147-52.

13. Xu D, Liu A, Wang X, et al. Repression of Septin9 and Septin2 suppresses tumor growth of human glioblastoma cells. *Cell Death Dis*. 2018;9(5):514.

14. Li W, He F. Monocyte to macrophage differentiation-associated (MMD) targeted by miR-140-5p regulates tumor growth in non-small cell lung cancer. *Biochem Biophys Res Commun*. 2014;450(1):844-50.

15. Tan X, Banerjee P, Guo HF, et al. Epithelial-to-mesenchymal transition drives a pro-metastatic Golgi compaction process through scaffolding protein PAQR11. *J Clin Invest*. 2017;127(1):117-131.

16. Jin T, Ding Q, Huang H, et al. PAQR10 and PAQR11 mediate Ras signaling in the Golgi apparatus. *Cell Res*. 2012;22(4):661-76.

17. Lin Y, Huang M, Wang S, You X, Zhang L, Chen Y. PAQR11 modulates monocyte-to-macrophage differentiation and pathogenesis of rheumatoid arthritis. *Immunology*. 2021;

18. Cao C, Lin S, Zhi W, et al. LOXL2 Expression Status Is Correlated With Molecular Characterizations of Cervical Carcinoma and Associated With Poor Cancer Survival via Epithelial-Mesenchymal Transition (EMT) Phenotype. *Front Oncol*. 2020;10:284.

19. Nguyen EV, Pereira BA, Lawrence MG, et al. Proteomic Profiling of Human Prostate Cancer-associated Fibroblasts (CAF) Reveals LOXL2-dependent Regulation of the Tumor Microenvironment. *Mol Cell Proteomics*. 2019;18(7):1410-1427.

20. Xie P, Yu H, Wang F, Yan F, He X. Inhibition of LOXL2 Enhances the Radiosensitivity of Castration-Resistant Prostate Cancer Cells Associated with the Reversal of the EMT Process. *Biomed Res Int*. 2019;2019:4012590.

21. Cebria-Costa JP, Pascual-Reguant L, Gonzalez-Perez A, et al. LOXL2-mediated H3K4 oxidation reduces chromatin accessibility in triple-negative breast cancer cells. *Oncogene*. 2020;39(1):79-121.

22. Park JS, Lee JH, Lee YS, Kim JK, Dong SM, Yoon DS. Emerging role of LOXL2 in the promotion of pancreas cancer metastasis. *Oncotarget*. 2016;7(27):42539-42552.

23. Park PG, Jo SJ, Kim MJ, et al. Role of LOXL2 in the epithelial-mesenchymal transition and colorectal cancer metastasis. *Oncotarget*. 2017;8(46):80325-80335.

24. Wang M, Zhao X, Zhu D, et al. HIF-1alpha promoted vasculogenic mimicry formation in hepatocellular carcinoma through LOXL2 up-regulation in hypoxic tumor microenvironment. *J Exp Clin Cancer Res*. 2017;36(1):60.

25. Matsuoka K, Bakiri L, Wolff LI, et al. Wnt signaling and Loxl2 promote aggressive osteosarcoma. *Cell Res*. 2020;30(10):885-901.

26. Xiang Z, Li J, Song S, et al. A positive feedback between IDO1 metabolite and COL12A1 via MAPK pathway to promote gastric cancer metastasis. *J Exp Clin Cancer Res*. 2019;38(1):314.

27. Tian J, Sun HX, Li YC, Jiang L, Zhang SL, Hao Q. LOXL 2 Promotes The Epithelial-Mesenchymal Transition And Malignant Progression Of Cervical Cancer. *Onco Targets Ther*. 2019;12:8947-8954.

28. Almacellas-Rabaiget O, Monaco P, Huertas-Martinez J, et al. LOXL2 promotes oncogenic progression in alveolar rhabdomyosarcoma independently of its catalytic activity. *Cancer Lett*. 2020;474:1-14.

29. Liu C, Guo T, Sakai A, et al. A novel splice variant of LOXL2 promotes progression of human papillomavirus-negative head and neck squamous cell carcinoma. *Cancer*. 2020;126(4):737-748.

30. Bergeat D, Fautrel A, Turlin B, et al. Impact of stroma LOXL2 overexpression on the prognosis of intrahepatic cholangiocarcinoma. *J Surg Res*. 2016;203(2):441-50.

31. Fossey SL, Bear MD, Kisseberth WC, Pennell M, London CA. Oncostatin M promotes STAT3 activation, VEGF production, and invasion in osteosarcoma cell lines. *BMC Cancer*. 2011;11:125.

32. Sharanek A, Burban A, Laaper M, et al. OSMR controls glioma stem cell respiration and confers resistance of glioblastoma to ionizing radiation. *Nat Commun*. 2020;11(1):4116.

33. Yu Z, Li Z, Wang C, et al. Oncostatin M receptor, positively regulated by SP1, promotes gastric cancer growth and metastasis upon treatment with Oncostatin M. *Gastric Cancer*. 2019;22(5):955-966.

34. Guo Q, Guan GF, Cao JY, et al. Overexpression of oncostatin M receptor regulates local immune response in glioblastoma. *J Cell Physiol*. 2019;

35. Shien K, Papadimitrakopoulou VA, Ruder D, et al. JAK1/STAT3 Activation through a Proinflammatory Cytokine Pathway Leads to Resistance to Molecularly Targeted Therapy in Non-Small Cell Lung Cancer. *Mol Cancer Ther*. 2017;16(10):2234-2245.

36. Hibi K, Goto T, Sakuraba K, et al. Methylation of OSMR gene is frequently observed in non-invasive colorectal cancer. *Anticancer Res*. 2011;31(4):1293-5.

37. Deng S, He SY, Zhao P, Zhang P. The role of oncostatin M receptor gene polymorphisms in bladder cancer. *World J Surg Oncol*. 2019;17(1):30.

38. Zhu YX, Li CH, Li G, et al. LLGL1 Regulates Gemcitabine Resistance by Modulating the ERK-SP1-OSMR Pathway in Pancreatic Ductal Adenocarcinoma. *Cell Mol Gastroenterol Hepatol*. 2020;10(4):811-828.

39. Kucia-Tran JA, Tulkki V, Scarpini CG, et al. Anti-oncostatin M antibody inhibits the pro-malignant effects of oncostatin M receptor overexpression in squamous cell carcinoma. *J Pathol*. 2018;244(3):283-295.

40. Qi XP, Zhao JQ, Chen ZG, et al. RET mutation p.S891A in a Chinese family with familial medullary thyroid carcinoma and associated cutaneous amyloidosis binding OSMR variant p.G513D. *Oncotarget*. 2015;6(32):33993-4003.

41. West NR, Murray JI, Watson PH. Oncostatin-M promotes phenotypic changes associated with mesenchymal and stem cell-like differentiation in breast cancer. *Oncogene*. 2014;33(12):1485-94.

42. Walker EC, McGregor NE, Poulton IJ, et al. Oncostatin M promotes bone formation independently of resorption when signaling through leukemia inhibitory factor receptor in mice. *J Clin Invest*. 2010;120(2):582-92.

43. David E, Tirode F, Baud'huin M, et al. Oncostatin M is a growth factor for Ewing sarcoma. *Am J Pathol*. 2012;181(5):1782-95.

44. Savarese TM, Campbell CL, McQuain C, et al. Coexpression of oncostatin M and its receptors and evidence for STAT3 activation in human ovarian carcinomas. *Cytokine*. 2002;17(6):324-34.

45. Goicoechea SM, Garcia-Mata R, Staub J, et al. Palladin promotes invasion of pancreatic cancer cells by enhancing invadopodia formation in cancer-associated fibroblasts. *Oncogene*. 2014;33(10):1265-73.

46. Chin YR, Toker A. Akt isoform-specific signaling in breast cancer: uncovering an anti-migratory role for palladin. *Cell Adh Migr*. 2011;5(3):211-4.

47. Sun HM, Chen XL, Chen XJ, et al. PALLD Regulates Phagocytosis by Enabling Timely Actin Polymerization and Depolymerization. *J Immunol*. 2017;199(5):1817-1826.

48. Tay PN, Tan P, Lan Y, et al. Palladin, an actin-associated protein, is required for adherens junction formation and intercellular adhesion in HCT116 colorectal cancer cells. *Int J Oncol*. 2010;37(4):909-26.

49. Gupta V, Bassi DE, Simons JD, et al. Elevated expression of stromal palladin predicts poor clinical outcome in renal cell carcinoma. *PLoS One*. 2011;6(6):e21494.

50. Cabibbo A, Pagani M, Fabbri M, et al. ERO1-L, a human protein that favors disulfide bond formation in the endoplasmic reticulum. *J Biol Chem*. 2000;275(7):4827-33.

51. Han F, Xu Q, Zhao J, Xiong P, Liu J. ERO1L promotes pancreatic cancer cell progression through activating the Wnt/catenin pathway. *J Cell Biochem*. 2018;119(11):8996-9005.

52. Lei Y, Zang R, Lu Z, et al. ERO1L promotes IL6/sIL6R signaling and regulates MUC16 expression to promote CA125 secretion and the metastasis of lung cancer cells. *Cell Death Dis*. 2020;11(10):853.

53. Shi X, Wu J, Liu Y, Jiang Y, Zhi C, Li J. ERO1L promotes NSCLC development by modulating cell cycle-related molecules. *Cell Biol Int*. 2020;44(12):2473-2484.

54. Li X, Li Y, Jiang C, Chen L, Gan N. MicroRNA-144-3p Inhibits Tumorigenesis of Oral Squamous Cell Carcinoma by downregulating ERO1L. *J Cancer*. 2020;11(3):759-768.

55. Zhou B, Wang G, Gao S, et al. Expression of ERO1L in gastric cancer and its association with patient prognosis. *Exp Ther Med*. 2017;14(3):2298-2302.

56. Hayes KE, Batsomboon P, Chen WC, et al. Inhibition of the FAD containing ER oxidoreductin 1 (Ero1) protein by EN-460 as a strategy for treatment of multiple myeloma. *Bioorg Med Chem*. 2019;27(8):1479-1488.

57. Sun K, He SB, Yao YZ, et al. Tre2 (USP6NL) promotes colorectal cancer cell proliferation via Wnt/beta-catenin pathway. *Cancer Cell Int*. 2019;19:102.

58. Ma T, Liu H, Liu Y, et al. USP6NL mediated by LINC00689/miR-142-3p promotes the development of triple-negative breast cancer. *BMC Cancer*. 2020;20(1):998.

59. Jun GR, Chung J, Mez J, et al. Transethnic genome-wide scan identifies novel Alzheimer's disease loci. *Alzheimers Dement*. 2017;13(7):727-738.

60. Licata LA, Hostetter CL, Crismale J, Sheth A, Keen JC. The RNA-binding protein HuR regulates GATA3 mRNA stability in human breast cancer cell lines. *Breast Cancer Res Treat*. 2010;122(1):55-63.

61. Winkler C, Doller A, Imre G, et al. Attenuation of the ELAV1-like protein HuR sensitizes adenocarcinoma cells to the intrinsic apoptotic pathway by increasing the translation of caspase-2L. *Cell Death Dis*. 2014;5:e1321.

62. Lal S, Burkhart RA, Beeharry N, et al. HuR posttranscriptionally regulates WEE1: implications for the DNA damage response in pancreatic cancer cells. *Cancer Res*. 2014;74(4):1128-40.

63. Zhang F, Cai Z, Lv H, et al. Multiple functions of HuR in urinary tumors. *J Cancer Res Clin Oncol*. 2019;145(1):11-18.

64. Farzan M, Schnitzler CE, Vasilieva N, Leung D, Choe H. BACE2, a beta -secretase homolog, cleaves at the beta site and within the amyloid-beta region of the amyloid-beta precursor protein. *Proc Natl Acad Sci U S A*. 2000;97(17):9712-7.

65. Stockley JH, O'Neill C. The proteins BACE1 and BACE2 and beta-secretase activity in normal and Alzheimer's disease brain. *Biochem Soc Trans*. 2007;35(Pt 3):574-6.

66. Southan C. BACE2 as a new diabetes target: a patent review (2010 - 2012). *Expert Opin Ther Pat*. 2013;23(5):649-63.

67. Yuan J, Zeng J, Shuai C, Liu Y. TWSG1 Is a Novel Tumor Suppressor in Gastric Cancer. *DNA Cell Biol*. 2018;37(6):574-583.

68. Xia S, Ji R, Xu Y, Ni X, Dong Y, Zhan W. Twisted Gastrulation BMP Signaling Modulator 1 Regulates Papillary Thyroid Cancer Cell Motility and Proliferation. *J Cancer*. 2017;8(14):2816-2827.

69. Christe M, Jin N, Wang X, et al. Transgenic mice with cardiac-specific over-expression of MLK7 have increased mortality when exposed to chronic beta-adrenergic stimulation. *J Mol Cell Cardiol*. 2004;37(3):705-15.

70. Hsieh YL, Tsai YL, Shibu MA, et al. ZAK induces cardiomyocyte hypertrophy and brain natriuretic peptide expression via p38/JNK signaling and GATA4/c-Jun transcriptional factor activation. *Mol Cell Biochem*. 2015;405(1-2):1-9.

71. Cheng YC, Kuo WW, Wu HC, et al. ZAK induces MMP-2 activity via JNK/p38 signals and reduces MMP-9 activity by increasing TIMP-1/2 expression in H9c2 cardiomyoblast cells. *Mol Cell Biochem*. 2009;325(1-2):69-77.

72. Nyati S, Chator A, Schinske K, Gregg BS, Ross BD, Rehemtulla A. A Requirement for ZAK Kinase Activity in Canonical TGF-beta Signaling. *Transl Oncol*. 2016;9(6):473-481.

73. Korkina O, Dong Z, Marullo A, Warshaw G, Symons M, Ruggieri R. The MLK-related kinase (MRK) is a novel RhoC effector that mediates lysophosphatidic acid (LPA)-stimulated tumor cell invasion. *J Biol Chem*. 2013;288(8):5364-73.

74. Markowitz D, Powell C, Tran NL, et al. Pharmacological Inhibition of the Protein Kinase MRK/ZAK Radiosensitizes Medulloblastoma. *Mol Cancer Ther*. 2016;15(8):1799-808.

75. Wang X, Mader MM, Toth JE, et al. Complete inhibition of anisomycin and UV radiation but not cytokine induced JNK and p38 activation by an aryl-substituted dihydropyrrolopyrazole quinoline and mixed lineage kinase 7 small interfering RNA. *J Biol Chem*. 2005;280(19):19298-305.

76. Jiang YX, Du ZM, Jiao L, et al. Inhibition of MiR-155 suppresses cell migration in nasopharyngeal carcinoma through targeting ZDHHC2. *Int J Clin Exp Med*. 2015;8(6):8472-84.

77. Peng C, Zhang Z, Wu J, et al. A critical role for ZDHHC2 in metastasis and recurrence in human hepatocellular carcinoma. *Biomed Res Int*. 2014;2014:832712.

78. Yan SM, Tang JJ, Huang CY, et al. Reduced expression of ZDHHC2 is associated with lymph node metastasis and poor prognosis in gastric adenocarcinoma. *PLoS One*. 2013;8(2):e56366.

79. Liu Z, Liu C, Xiao M, Han Y, Zhang S, Xu B. Bioinformatics Analysis of the Prognostic and Biological Significance of ZDHHC-Protein Acyltransferases in Kidney Renal Clear Cell Carcinoma. *Front Oncol*. 2020;10:565414.

80. Li Y, Xu J, Xiong H, et al. Cancer driver candidate genes AVL9, DENND5A and NUPL1 contribute to MDCK cystogenesis. *Oncoscience*. 2014;1(12):854-865.

81. Tang J, Li Y, Lyon K, et al. Cancer driver-passenger distinction via sporadic human and dog cancer comparison: a proof-of-principle study with colorectal cancer. *Oncogene*. 2014;33(7):814-22.

82. Lukas J, Mazna P, Valenta T, et al. Dazap2 modulates transcription driven by the Wnt effector TCF-4. *Nucleic Acids Res*. 2009;37(9):3007-20.

83. Roche DD, Liu KJ, Harland RM, Monsoro-Burq AH. Dazap2 is required for FGF-mediated posterior neural patterning, independent of Wnt and Cdx function. *Dev Biol*. 2009;333(1):26-36.

84. Sugawara T, Miura T, Kawasaki T, Umezawa A, Akutsu H. The hsa-miR-302 cluster controls ectodermal differentiation of human pluripotent stem cell via repression of DAZAP2. *Regen Ther*. 2020;15:1-9.

85. Li J, Hu WX, Luo SQ, et al. Promoter methylation induced epigenetic silencing of DAZAP2, a downstream effector of p38/MAPK pathway, in multiple myeloma cells. *Cell Signal*. 2019;60:136-145.

86. Zhang T, Zheng T, Wang C, et al. EFFECTS OF Wnt / beta-CATENIN SIGNALING PATHWAY AND STAR D7 ON TESTOSTERONE SYNTHESIS. *Acta Endocrinol (Buchar)*. 2018;14(2):155-162.

87. Flores-Martin J, Rena V, Marquez S, Panzetta-Dutari GM, Genti-Raimondi S. StarD7 knockdown modulates ABCG2 expression, cell migration, proliferation, and differentiation of human choriocarcinoma JEG-3 cells. *PLoS One*. 2012;7(8):e44152.

88. Durand S, Angeletti S, Genti-Raimondi S. GTT1/StarD7, a novel phosphatidylcholine transfer protein-like highly expressed in gestational trophoblastic tumour: cloning and characterization. *Placenta*. 2004;25(1):37-44.

89. Lee JH, Horak CE, Khanna C, et al. Alterations in Gemin5 expression contribute to alternative mRNA splicing patterns and tumor cell motility. *Cancer Res*. 2008;68(3):639-44.

90. Pan H, Pan J, Ji L, et al. Carboxypeptidase A4 promotes cell growth via activating STAT3 and ERK signaling pathways and predicts a poor prognosis in colorectal cancer. *Int J Biol Macromol*. 2019;138:125-134.

91. Zhang H, Hao C, Wang H, Shang H, Li Z. Carboxypeptidase A4 promotes proliferation and stem cell characteristics of hepatocellular carcinoma. *Int J Exp Pathol*. 2019;100(2):133-138.

92. Shao Q, Zhang Z, Cao R, Zang H, Pei W, Sun T. CPA4 Promotes EMT in Pancreatic Cancer via Stimulating PI3K-AKT-mTOR Signaling. *Onco Targets Ther*. 2020;13:8567-8580.

93. Sun L, Wang Y, Yuan H, et al. CPA4 is a Novel Diagnostic and Prognostic Marker for Human Non-Small-Cell Lung Cancer. *J Cancer*. 2016;7(10):1197-204.

94. Bademler S, Ucuncu MZ, Tilgen Vatansever C, Serilmez M, Ertin H, Karanlik H. Diagnostic and Prognostic Significance of Carboxypeptidase A4 (CPA4) in Breast Cancer. *Biomolecules*. 2019;9(3)

95. Huang Z, Li J, Du S, et al. FKBP14 overexpression contributes to osteosarcoma carcinogenesis and indicates poor survival outcome. *Oncotarget*. 2016;7(26):39872-39884.

96. Wang R, Fang H, Fang Q. Downregulation of FKBP14 by RNA interference inhibits the proliferation, adhesion and invasion of gastric cancer cells. *Oncol Lett*. 2017;13(4):2811-2816.

97. Lu M, Miao Y, Qi L, Bai M, Zhang J, Feng Y. RNAi-Mediated Downregulation of FKBP14 Suppresses the Growth of Human Ovarian Cancer Cells. *Oncol Res*. 2016;23(6):267-274.

98. Worley BL, Auen T, Arnold AC, Monia BP, Hempel N, Czyzyk TA. Antisense oligonucleotide-mediated knockdown of Mpzl3 attenuates the negative metabolic effects of diet-induced obesity in mice. *Physiol Rep*. 2021;9(9):e14853.

99. Racz P, Mink M, Ordas A, et al. The human orthologue of murine Mpzl3 with predicted adhesive and immune functions is a potential candidate gene for immune-related hereditary hair loss. *Exp Dermatol*. 2009;18(3):261-3.

100. Bhaduri A, Ungewickell A, Boxer LD, Lopez-Pajares V, Zarnegar BJ, Khavari PA. Network Analysis Identifies Mitochondrial Regulation of Epidermal Differentiation by MPZL3 and FDXR. *Dev Cell*. 2015;35(4):444-57.

101. Nicu C, Wikramanayake TC, Paus R. Clues that mitochondria are involved in the hair cycle clock: MPZL3 regulates entry into and progression of murine hair follicle cycling. *Exp Dermatol*. 2020;29(12):1243-1249.

102. Yokoyama-Mashima S, Yogosawa S, Kanegae Y, et al. Forced expression of DYRK2 exerts anti-tumor effects via apoptotic induction in liver cancer. *Cancer Lett*. 2019;451:100-109.

103. Kumamoto T, Yamada K, Yoshida S, et al. Impairment of DYRK2 by DNMT1mediated transcription augments carcinogenesis in human colorectal cancer. *Int J Oncol*. 2020;56(6):1529-1539.

104. Wang Y, Wu Y, Miao X, et al. Silencing of DYRK2 increases cell proliferation but reverses CAM-DR in Non-Hodgkin's Lymphoma. *Int J Biol Macromol*. 2015;81:809-17.

105. Mimoto R, Taira N, Takahashi H, et al. DYRK2 controls the epithelial-mesenchymal transition in breast cancer by degrading Snail. *Cancer Lett*. 2013;339(2):214-25.

106. Wang Y, Sun J, Wei X, et al. Decrease of miR-622 expression suppresses migration and invasion by targeting regulation of DYRK2 in colorectal cancer cells. *Onco Targets Ther*. 2017;10:1091-1100.

107. Maekawa M, Hiyoshi H, Nakayama J, et al. Cullin-3/KCTD10 complex is essential for K27-polyubiquitination of EIF3D in human hepatocellular carcinoma HepG2 cells. *Biochem Biophys Res Commun*. 2019;516(4):1116-1122.

108. Murakami A, Maekawa M, Kawai K, et al. Cullin-3/KCTD10 E3 complex is essential for Rac1 activation through RhoB degradation in human epidermal growth factor receptor 2-positive breast cancer cells. *Cancer Sci*. 2019;110(2):650-661.

109. Kubota D, Yoshida A, Tsuda H, et al. Gene expression network analysis of ETV1 reveals KCTD10 as a novel prognostic biomarker in gastrointestinal stromal tumor. *PLoS One*. 2013;8(8):e73896.

110. Huang Z, Cai Y, Yang C, et al. Knockdown of RNF6 inhibits gastric cancer cell growth by suppressing STAT3 signaling. *Onco Targets Ther*. 2018;11:6579-6587.

111. Huang ZM, Ge HF, Yang CC, et al. MicroRNA-26a-5p inhibits breast cancer cell growth by suppressing RNF6 expression. *Kaohsiung J Med Sci*. 2019;35(8):467-473.

112. Liu L, Zhang Y, Wong CC, et al. RNF6 Promotes Colorectal Cancer by Activating the Wnt/beta-Catenin Pathway via Ubiquitination of TLE3. *Cancer Res*. 2018;78(8):1958-1971.

**Table S2. Hypoxic regulation of the 16 common KDM4B targets in the three cell lines.**

| **Gene** | **Protein name** | **Oxygen condition** | **Regulation** | **Log2 ratio** | | **P value** | **FDR** | **Sample type** | | **Evidence level** | **Method** |
| --- | --- | --- | --- | --- | --- | --- | --- | --- | --- | --- | --- |
| *MMD* | monocyte to macrophage differentiation associated | hypoxia/24h vs normoxia | ↑ | 1.049 | | <0.001 | 0.014 | HCT116 cells | | transcript | high throughput sequencing |
| *OSMR* | oncostatin M receptor | hypoxia/24h vs normoxia | ↑ | 1.003 | | <0.001 | 0.01 | HCT116 cells | | transcript | high throughput sequencing |
|  |  | hypoxia/24h vs normoxia | ↑ | 1.451 | | <0.001 | 0.001 | HCT116 cells | | transcript | high throughput sequencing |
|  |  | hypoxia/24h vs normoxia | ↑ | 1.857 | | <0.001 | 0.007 | HCT116 cells | | transcript | high throughput sequencing |
|  |  | 0%/48h vs normoxia | ↑ | 2.357 | | <0.001 | 0.05 | HCCT116 cells | | transcript | array |
| *PALLD* | palladin, cytoskeletal associated protein | 1%/48h vs normoxia | ↓ | -0.273 | | N/A | N/A | 786-O/VHL cells | | protein | SILAC |
|  |  | 1%/24h vs normoxia | ↑ | 0.172 | | N/A | N/A | HCT116 cells | | protein | SILAC |
| *USP6NL* | USP6 N-terminal like | 1%/24h vs normoxia | ↓ | -0.086 | | N/A | N/A | HCT116 cells | | protein | SILAC |
|  |  | 0%/48h vs normoxia | ↓ | -2.211 | | <0.001 | 0.004 | HCCT116 cell | | transcript | array |
| *DAZAP2* | DAZ associated protein 2 | hypoxia vs normoxia | ↑ | 1.02 | | <0.001 | 0.004 | HT29 cells | | transcript | microarray analysis |
| *ERO1L* | ERO1-Like (S. Cerevisiae) | 1%/24h vs normoxia* |  | N/A | |  |  | HCT116 cells* | | Protein* | Immunoblot* |
|  |  | 1%/24h vs normoxia | ↑ | 1.09 | | N/A | N/A | HCT116 cells | | protein | SILAC |
| **Gene** | **Protein name** | **Condition** | **Regulaion** | **Log2 ratio** | | **P value** | **FDR** | **Sample type** | | **Evidence level** | **Method** |
| *SEPT2* | septin 2 | 1%/24h vs normoxia | ↑ | 0.179 | | N/A | N/A | HCT116 cells | | protein | SILAC |
|  |  | hypoxia/24h vs normoxia | ↑ | 1.378 | | 0.002 | 0.043 | HCT116 cells | | transcript | high throughput sequencing |
|  |  | 1%/24h vs normoxia | ↓ | -1.105 | | N/A | N/A | HCT116 cells (secretome) | | protein | SILAC |
|  |  | hypoxia/24h vs normoxia | ↓ | -1.185 | | 0.001 | 0.033 | HCT116 cells | | transcript | high throughput sequencing |
|  |  | 1%/48h vs normoxia | ↑ | 0.043 | | N/A | N/A | 786-O/VHL cells | | protein | SILAC |
| *LOXL2* | lysyl oxidase like 2 | hypoxia/24h vs normoxia | ↑ | 1.053 | | <0.001 | 0.009 | HCT116 cells | | transcript | high throughput sequencing |
|  |  | 1%/24h vs normoxia | ↑ | 1.104 | | N/A | N/A | renal proximal tubule epithelial cells | | transcriptomics | N/A |
|  |  | hypoxia/24h vs normoxia | ↑ | 1.262 | | <0.001 | <0.001 | HCT116 cells | | transcript | high throughput sequencing |
|  |  | 1%/24h vs normoxia | ↑ | 1.411 | | N/A | N/A | renal proximal tubule epithelial cells | | transcriptomics | N/A |
| **Gene** | **Protein name** | **Condition** | **Regulaion** | **Log2 ratio** | | **P value** | **FDR** | **Sample type** | | **Evidence level** | **Method** |
| *LOXL2* | lysyl oxidase like 2 | 1%/24h vs normoxia | ↑ | 1.428 | | N/A | N/A | HCT116 cells (secretome) | | protein | SILAC |
|  |  | 0%/48h vs normoxia | ↑ | 1.531 | | <0.001 | 0.003 | HCCT116 cell | | transcript | array |
|  |  | 1%/48h vs normoxia | ↑ | 1.548 | | <0.001 | <0.001 | ovarian cancer cell line A2780 | | transcript | high throughput sequencing |
|  |  | 1%/6d vs normoxia | ↑ | 2.405 | | <0.001 | <0.001 | ovarian cancer cell line A2780 | | transcript | high throughput sequencing |
|  |  | 1%/72h vs normoxia | ↑ | 3.819 | | <0.001 | <0.001 | SK-OV-3 ovarian cancer cells | | transcript | array |
|  |  | hypoxia/24h vs normoxia | ↑ | 4.283 | | <0.001 | <0.001 | HCT116 cells | | transcript | high throughput sequencing |
| *ELAV1* | ELAV like RNA binding protein 1 | 1%/24h vs normoxia | ↑ | 0.011 | N/A | | N/A | HCT116 cells | protein | | SILAC |
|  |  | 1%/48h vs normoxia | ↓ | -0.029 | N/A | | N/A | 786-O/VHL cells | protein | | SILAC |
|  |  | 1%/24h vs normoxia | ↓ | -0.467 | N/A | | N/A | HCT116 cells (secretome) | protein | | SILAC |

| **Gene** | **Protein name** | **Condition** | **Regulaion** | **Log2 ratio** | **P value** | **FDR** | **Sample type** | **Evidence level** | **Method** |
| --- | --- | --- | --- | --- | --- | --- | --- | --- | --- |
| *ZAK* | mitogen-activated protein kinase kinase kinase 20 | 1%/24h vs normoxia | ↑ | 0.196 | N/A | N/A | HCT116 cells | protein | SILAC |
| *DENND5A* | DENN domain containing 5A | hypoxia/24h vs normoxia | ↓ | -1.165 | <0.001 | 0.001 | HCT116 cells | transcript | high throughput sequencing |
| *DAZAP2* | DAZ associated protein 2 | hypoxia vs normoxia | ↑ | 1.02 | <0.001 | 0.004 | HT29 cells | transcript | microarray analysis |
| *LUZP6* | Leucine Zipper Protein 6 | 1%/24h vs normoxia | ↑ | 0.094 | N/A | N/A | HCT116 cells | protein | SILAC |

*The asterisk mark shows the evidence from low-throughput experiment

This table shows the information from the database of iHypoxia (integrative database for Hypoxia associated proteins in mammals, <http://ihypoxia.omicsbio.info/>), which host the results of hypoxia regulatory proteins based on low-throughput and high-throughput identification.
